# Model Steatogenic Compounds (Amiodarone, Valproic Acid, and Tetracycline) Alter Lipid Metabolism by Different Mechanisms in Mouse Liver Slices

**DOI:** 10.1371/journal.pone.0086795

**Published:** 2014-01-29

**Authors:** Ewa Szalowska, Bart van der Burg, Hai-Yen Man, Peter J. M. Hendriksen, Ad A. C. M. Peijnenburg

**Affiliations:** 1 Cluster of Bioassays and Toxicology, RIKILT - Institute of Food Safety, Wageningen University and Research Centre, Wageningen, The Netherlands; 2 BDS BioDetection Systems, Amsterdam, The Netherlands; Clermont Université, France

## Abstract

Although drug induced steatosis represents a mild type of hepatotoxicity it can progress into more severe non-alcoholic steatohepatitis. Current models used for safety assessment in drug development and chemical risk assessment do not accurately predict steatosis in humans. Therefore, new models need to be developed to screen compounds for steatogenic properties. We have studied the usefulness of mouse precision-cut liver slices (PCLS) as an alternative to animal testing to gain more insight into the mechanisms involved in the steatogenesis. To this end, PCLS were incubated 24 h with the model steatogenic compounds: amiodarone (AMI), valproic acid (VA), and tetracycline (TET). Transcriptome analysis using DNA microarrays was used to identify genes and processes affected by these compounds. AMI and VA upregulated lipid metabolism, whereas processes associated with extracellular matrix remodelling and inflammation were downregulated. TET downregulated mitochondrial functions, lipid metabolism, and fibrosis. Furthermore, on the basis of the transcriptomics data it was hypothesized that all three compounds affect peroxisome proliferator activated-receptor (PPAR) signaling. Application of PPAR reporter assays classified AMI and VA as PPARγ and triple PPARα/(β/δ)/γ agonist, respectively, whereas TET had no effect on any of the PPARs. Some of the differentially expressed genes were considered as potential candidate biomarkers to identify PPAR agonists (i.e. AMI and VA) or compounds impairing mitochondrial functions (i.e. TET). Finally, comparison of our findings with publicly available transcriptomics data showed that a number of processes altered in the mouse PCLS was also affected in mouse livers and human primary hepatocytes exposed to known PPAR agonists. Thus mouse PCLS are a valuable model to identify early mechanisms of action of compounds altering lipid metabolism.

## Introduction

Drug induced fatty liver (steatosis) belongs to one of the most common forms of liver injury [1]. Although benign steatosis does not severely affect liver function and is reversible, chronic exposure to steatogenic drugs could lead to the development of steatosis associated with inflammation, referred to as non-alcoholic steatohepatitis (NASH). Eventually, NASH can progress to irreversible liver diseases, including fibrosis, cirrhosis, and liver cancer requiring liver transplant [2]. To minimize the chances of developing steatosis and related liver disorders, compounds with steatogenic properties need to be identified during the early stages of drug development. In general, steatosis is characterized by accumulation of vacuoles filled with triglycerides (TG). The exact molecular triggers resulting in lipid accumulation in the liver are largely unknown, but may arise from: 1) increased uptake of lipids, 2) elevated *de novo* lipogenesis, 3) impaired lipoprotein synthesis and secretion, and/or 4) reduced catabolism of fatty acids (FA) by peroxisomal/mitochondrial β-oxidation [3]. One of the most common causes of drug-induced steatosis is impairment of mitochondrial functions. Mitochondria are essential for energy generation in the cell through FA β-oxidation, pyruvate oxidation, and adenosine triphosphate (ATP) synthesis by oxidative phosphorylation [4]. Mitochondrial β-oxidation is the major process that eliminates FA, which accumulate in a form of TG in liver cells if not-catabolised. Consistent with these notions, many steatogenic drugs interfere directly with enzymes involved in β-oxidation [4]. Drug-induced perturbations of mitochondrial membranes, transcripts or proteins involved in replication of its DNA could secondarily impair mitochondrial functions [4]. In addition, deregulation of lipid metabolism via interactions of drugs with key regulators of lipid homeostasis, exemplified by members of the nuclear receptor family such as pregnane X receptor (PXR), liver X receptor (LXR), or peroxisome proliferator activated receptors (PPARs), has been reported as well [5]. In particular, alterations in the expression of PPARα target genes involved in lipid catabolism (e.g. carnitine palmityltransferase 1 (*Cpt1*), 3-ketoacyl-CoA thiolase (*Hadhb*), acetyl-Coenzyme A acyltransferase 2 (*Acaa2*)), have been linked to the development of drug-induced steatosis [6].

With regard to known steatogenic drugs, the commonly used antibiotic, tetracycline (TET), inhibits FA catabolism in mice liver [7]–[9] and *in vitro* models, such as cultures of rat and dog hepatocytes [10]–[12]. Another example of a steatogenic drug is amiodarone (AMI). Although AMI is currently approved as an anti-arrhythmic agent [13], about 18% of patients discontinue AMI therapy due to undesirable side effects, including development of NASH [14]. The steatogenic actions of AMI are related to inhibition of mitochondrial β-oxidation, paradoxically associated with upregulation of PPARα target genes involved in lipid catabolism [15], [16]. Valproic acid (VA), next to its beneficial effects in treatment of epilepsy and bipolar disorder, has been implicated in drug-induced steatosis. The steatogenic actions of VA are mainly associated with perturbations in mitochondrial β-oxidation [17].

Regulation of lipid metabolism in the liver *in vivo* involves interaction of both parenchymal (hepatocytes) and non-parenchymal cells (e.g. Kupffer and stellate cells) [18]. Consistent with this notion, treatment of rat liver *in vivo* and primary hepatocytes *in vitro* with Ppar*α* agonists (fibrates) resulted in significant upregulation of genes involved in lipid metabolism in both systems. However, downregulation of genes involved in cellular morphogenesis, extracellular matrix remodelling, immune response and coagulation occurred only *in vivo* [19]. Therefore, application of mono-hepatocyte models to study lipid metabolism does not reflect the entire spectrum of responses characteristic for the liver *in vivo*. These shortcomings could be overcome by using precision cut liver slices (PCLS) that retain native liver architecture as well as both parenchymal and non-parenchymal cellular components [20].

In this study, mouse PCLS were used as an *in vitro* liver model to investigate mechanisms involved in drug- induced steatosis. The main goal was to validate PCLS as a tool to identify early mechanisms of action of three model steatogenic compounds: TET, AMI, and VA. Transcriptome analysis was combined with gene reporter assays to substantiate findings related to steatogenic properties of the selected compounds.

## Materials and Methods

### Chemicals

AMI, VA, TET, cyclosporin A (CsA), chlorpromazine (CPZ), ethinyl estradiol (EE), paraquat (PQ), isoniazid (ISND), acetaminophen (APAP) and bovine serum albumin (BSA) were purchased from Sigma (Sigma, Zwijndrecht, The Netherlands). Williams E medium (WEM) supplemented with Glutamax, penicillin/streptomycin (pen/strep), D-glucose, phosphate buffered saline (PBS) were obtained from Invitrogen (Invitrogen, Bleiswijk, The Netherlands). GW7647, rosiglitazone, and L165,041 were purchased from Cayman Chemical (Cayman Chemical, Ann Arbor, MI, USA). G418-disulfate was obtained from Duchefa Biochemie (Duchefa Biochemie, Haarlem, The Netherlands).

### Preparation and Culture of Liver Slices

Twenty three week-old male C57BL/6 mice from Harlan (Horst, The Netherlands) were housed for 1 week at 22°C with a relative humidity of 30–70%. The lighting cycle was 12-h light and 12-h dark. At 24 weeks, the animals were killed with an overdose of isoflurane, as approved by the Ethical Committee for Animal Experiments at Wageningen University. Immediately afterwards the livers were perfused with PBS and placed in ice-cold Krebs–Henseleit buffer (KHB) (pH 7.4, supplemented with 11 mM glucose). The tissue was transported to the laboratory within ∼30 min and cylindrical liver cores were produced with a surgical biopsy punch of 5 mm diameter (KAI, SynErgo Europe, Romania). The cores were placed in a Krumdieck tissue slicer (Alabama Research and Development, Munford, AL, USA) filled with ice-cold KHB aerated with carbogen and supplemented with 11 mM glucose. Slices 5 mm in diameter and 0.2 mm in thickness weighing ∼6 mg were prepared. Immediately afterwards, the slices were transferred to culture plates filled with WEM supplemented with pen/strep at 37°C. Three liver slices were pre-cultured in one well of the 6-well plate filled with 4 ml of WEM for 1 h with continuous shaking (70 rpm). An oxygen controlled incubator was used at 80% oxygen, 5% CO_2_ and the rest was N_2_. After 1 h pre-incubation, the medium was removed, refreshed, and supplemented with the test compounds or their appropriate solvents. After 24 h incubation, samples were snap-frozen in liquid nitrogen and stored in −80°C for later analysis. Samples for histology were fixed in 4% formaldehyde at room temperature.

### Cytotoxicity Analysis (Dose Selection)

**Figure 1:**
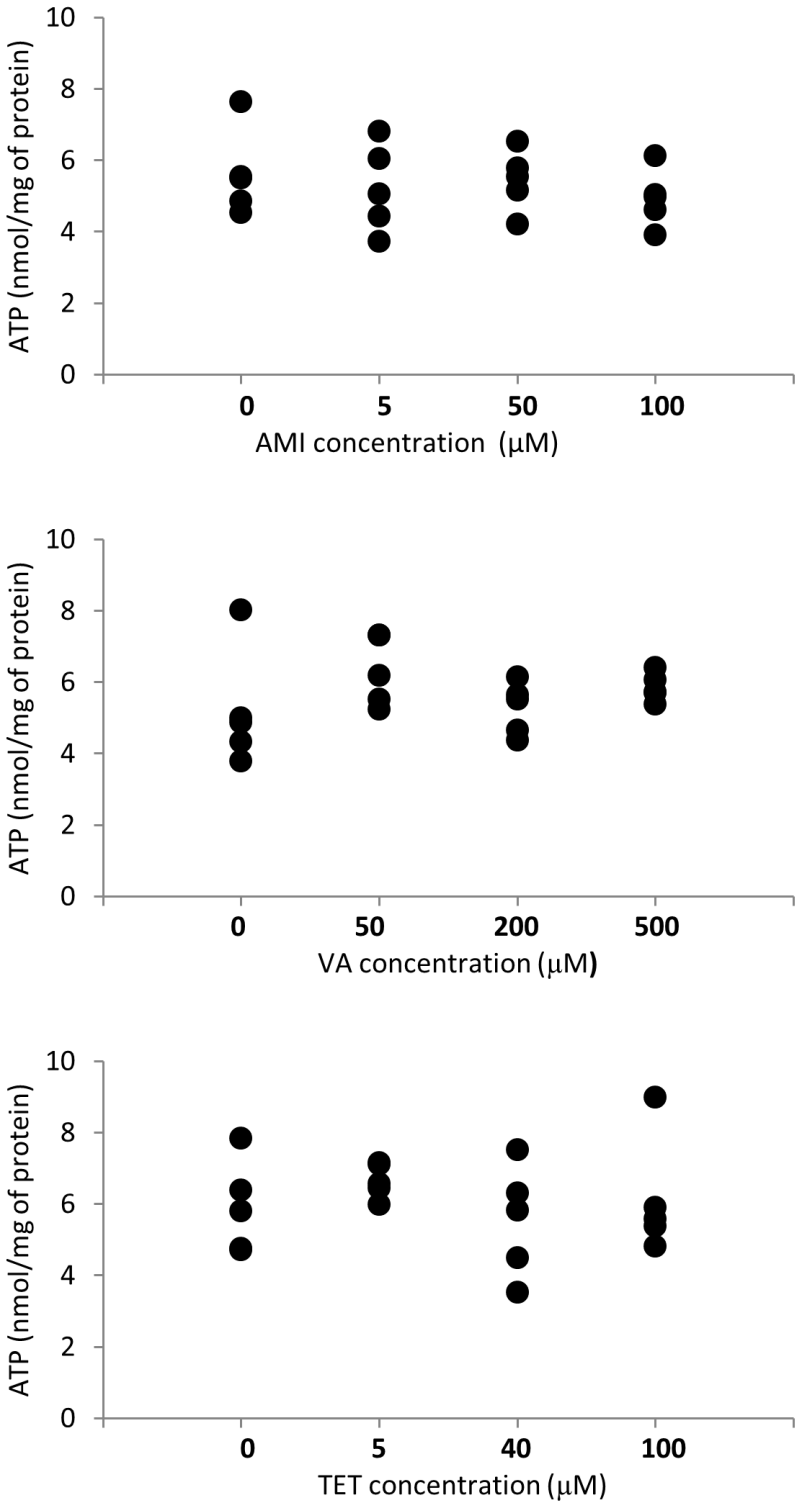
Viability of mouse liver slices upon treatment with steatogenic drugs. Liver slices were incubated for 24(AMI) 25, 50, and 100 µM, valproic acid (VA) 50, 200, and 500 µM, and tetracycline (TET) 5, 40, and 100 µM. ATP content (nmol/mg of protein) in slices treated with different concentrations of hepatotoxicants was compared to control slices. Each point is the mean±SD of 5 independent experiments (liver slices were isolated from livers of 5 mice) and each measurement was made in duplicate. There were no significant differences between the tested conditions.

PCLS were exposed to the different compounds inducing steatosis, cholestasis, and necrosis, which had been selected based on published reports. The steatogenic compounds were AMI, VA, and TET [7], [13], [17], the cholestatic compounds were represented by CsA, CPZ, and EE [21]–[23]. As necrotic agents, PQ, ISND, and APAP were used [24]–[26]. To find a non-toxic dose for subsequent gene expression profiling experiments, the tested concentration ranges were: CsA 0–100 µM, CPZ 0–80 µM, EE 0–100 µM, AMI 0–100 µM, VA 0–500 µM, TET 0–100 µM, PQ 0–10 µM, APAP 0–3000 µM and ISND 0–1000 µM. CsA, CPZ, AMI, PQ, and EE were dissolved in DMSO, VA and TET were dissolved in ethanol (EtOH), and ISND was dissolved in PBS. The compounds were added to the culture medium at 0.1% vol/vol in an appropriate solvent (DMSO, EtOH, or PBS). Slices incubated with the solvents at 0.1% vol/vol served as controls. The viability of the slices was assessed by measuring their ATP content (see below). Doses for the 3 steatogenic compounds were selected based on 5 independent experiments performed in slices obtained from livers of 5 mice (Figure 1). Doses for cholestatic and necrotic drugs were tested in liver slices obtained from 2 mice (Figure S1) and concentrations that did not decrease the level of ATP normalized to protein values compared to controls were selected for final exposure experiments. The selected concentrations for cholestatic and necrotic drugs were tested again in liver slices obtained from 5 different mice to confirm that they were non-toxic, Figure S2.

### ATP and Protein Measurement

For each ATP and protein measurement a total of 3 co-cultured slices were placed in 400 µL Cell Lytic MT buffer (Sigma, Zwijndrecht, the Netherlands). These were homogenized twice (15 sec, 6500 *g,* 8°C) using a tissue homogenizer Precellys 24 Bertin Technologies (Labmakelaar Benelux B.V. Rotterdam, The Netherlands). To remove cellular debris, the homogenates were centrifuged for 5 min (14000 *g*, 8°C) and the remaining supernatant was divided into 2 portions of 200 µL. One portion was stored at −80°C for protein measurement and the second 200 µL portion was mixed with 100 µL of ATP lytic buffer from ATPlite kit (Perkin Elmer, Oosterhout, The Netherlands) for ATP measurement, which was carried out with a microplate reader Synergy TM HT Multi Detection Microplate Reader (Biotek Instruments Inc, Abcoude, the Netherlands) with settings for luminescence: 590/635 nm, top measurement, and sensitivity 230. ATP was determined in technical duplicates and luminescence values were recalculated as µM ATP in total liver slice extracts.

Protein concentration was determined by the Bradford method protein assay (BioRad, Veenendaal, The Netherlands). Protein samples of 2 µL were diluted 80 times in PBS and measured, with BSA used as a standard, each measurement being taken in duplicate. ATP concentration was normalized to mg of protein per slice.

### PCLS Exposure (Gene Expression Profiling)

For transcriptome analysis, PCLS were cultured in the same conditions as above. Slices were exposed for 24 h to each concentration of the tested compounds or controls. The concentrations used were as follows; for the steatotic exposures: 50 µM AMI, 200 µM VA, and TET 40 µM. For the cholestatic exposures: 40 µM CsA, 20 µM CPZ, and 10 µM EE. For the necrotic compounds: 1000 µM APAP, 1000 µM ISND, and 5 µM PQ. PCLS obtained from 5 mice were used in 5 separate experiments in which exposure to toxic compound or vehicle were done simultaneously.

### DNA Microarray Hybridizations

Gene expression analysis in PCLS incubated for 24 h was done on HT Mouse Genome 430 PM array plates using the Affymetrix GeneTitan system (Affymetrix, Santa Clara, CA, USA). RNA was extracted from 3 slices cultured and exposed together using the RNeasy Tissue Mini Kit (Qiagen, Venlo, The Netherlands). RNA concentration and purity were assessed spectrometrically using a Nano Drop ND-1000 spectrophotometer (Isogen, IJsselstein, The Netherlands) by measuring absorption ratios at 260/280 and 230/280 nm. The integrity of the RNA samples was examined using the Shimadzu MultiNA Bioanalyzer (Shimadzu, Tokyo, Japan). Biotin- labelled cRNA was generated from high-quality total RNA with the Affymetrix 3′IVT Express Kit with an input of 100 ng total RNA. The Agilent Bioanalyzer (Agilent, Amstelveen, the Netherlands) and the Shimadzu MultiNA Bioanalyzer (Shimadzu,Tokyo, Japan) were used to assess the quality of cRNA in order to confirm if the average fragment size was in accordance with the Affymetrix specifications. Per sample, 7.5 ug cRNA of the biotinylated cRNA samples was fragmented and hybridized at 0.037 ug/ul on the Affymetrix HT Mouse genome 430 PM arrays. After automated washing and staining by a GeneTitan machine (Affymetrix, Santa Clara, CA, USA) using the Affymetrix HWS kit for Gene Titan, absolute values of expression were calculated from the scanned array using Affymetrix Command Console v 3.2 software. Data Quality Control was checked with the program Affymetrix Expression Console v 1.1 software to determine if all parameters were within quality specifications. The Probe Logarithmic Intensity Error Estimation (PLIER) algorithm method was used for probe summarisation [27].

In order to monitor the sample-independent control and the performance of each individual sample during hybridization, controls were added to the hybridization mixture. The sample-dependent controls, such as internal control genes, background values, and average signals, were used to determine the biological variation between samples. In conclusion, all the data were within the data Quality Control thresholds, according to Affymetrix Expression Console specifications. Non-normalized data in a form of the Cell Intensity File (*.CEL) were re-annotated (EntrezGene htmg430 pm Mm ENTREZG) and the data were RMA normalized [27].

All microarray datasets were deposited to Gene Expression Omnibus (GEO). The GEO series accession numbers are as follows: GSE51545 (contain all data used in our study). The GEO sub-series accession numbers are: GSE51543 (exposures to the steatogenic compounds), GSE51544 (exposures to the cholestatic compounds), and GSE51542 (exposures to the necrotic compounds).

### Gene Set Enrichment Analysis (GSEA)

To identify differentially expressed gene sets related to diverse biological functions, Gene Set Enrichment Analysis (GSEA) was performed with an open access bioinformatics tool (http://www.broadinstitute.org/gsea/index.jsp). In short, this method identifies biologically and functionally related genes affected due to experimental conditions. GSEA applies predefined gene sets based on the literature or other experiments. Gene sets contain a group of genes specific for a certain biological process, gene ontology (GO), pathway, or user defined group. GSEA ranks all the genes on their expression ratios between a treatment and the control group, and determines whether a particular gene set is significantly enriched at the top or the bottom of the ranked list [28]. Gene sets with p<0.05, FDR<0.05 were considered as significant. Gene sets used in this study were created in an open access bioinformatics tool ANNI http://www.biosemantics.org/index.php?page=ANNI-2-0 [29]. ANNI retrieves all the information available on known gene-gene associations present in Medline and can be used, among others, to create gene sets associated with simple queries, for example “inflammation” or “cholestasis”. For the purpose of this study, we used several queries related to liver specific and non-specific processes. A summary of the queries used for the creation of the ANNI gene sets is given in Table S1. Genes present in at least 5 publications indicating an association with the specified queries were included in the ANNI gene sets.

Gene sets called “Wy14643 acute” (i.e. 6 hours exposure in mouse liver *in vivo*) and “Wy14643 chronic” (i.e. 5 days exposure in mouse liver *in vivo*) were also used. These gene sets were derived from data deposited at Gene Expression Omnibus (GEO): GSE8292 (http://www.ncbi.nlm.nih.gov/geo/query/acc.cgi?acc=GSE8292) and GSE8295 (http://www.ncbi.nlm.nih.gov/geo/query/acc.cgi?acc=GSE8295), respectively. Genes present in these gene sets were selected based on analysis done in an open access bioinformatics tool, Bioconductor 2.12, using Linear Models for Microarray Data (LIMMA) [30]. A false discovery rate (FDR) q-value<0.05 and absolute fold change (FC) above 1.6 were applied for identification of significant genes.

For GSEA, GEO microarray data relevant for actions of known PPAR agonists in mouse liver *in vivo* and human primary hepatocytes were used. To study the effects of PPARs’ agonists in mouse *in vivo* following data sets were used: GSE32706 (fenofibrate and fish oil treatments for 14 days; http://www.ncbi.nlm.nih.gov/geo/query/acc.cgi?acc=GSE32706) and GSE8295 (Wy14673 chronic (5 days) exposure, http://0-www.ncbi.nlm.nih.gov.elis.tmu.edu. tw/geo/query/acc.cgi?acc = GSE8295). For the action of PPARs’ agonists in human primary hepatocytes data sets such as GSE33152 (dual PPARα/γ agonists treatment for 6 h, http://www.ncbi.nlm.nih.gov/geo/query/acc.cgi?acc=GSE33152) and GSE17251 (Wy14643 treatment; http://www.ncbi.nlm.nih.gov/geo/query/acc.cgi?acc=GSE17251) were used.

### Gene Functional Classification Analysis

The GSEA report output file informs which gene sets are significantly affected in the analysed experimental groups based on the enrichment at the top or the bottom of the ranked list of genes detected on a microarray [28]. In addition, it informs, which genes in the identified significant gene sets, contribute to this enrichment based on their ranking position. Thus only genes from the identified significant gene sets, which are found at the top or at the bottom of the ranked list, will be assigned by GSEA as genes contributing to the significant enrichment in the tested gene sets. Therefore genes, which are not located at the top or the bottom of the ranked list, are not considered by GSEA as genes that contribute to the significant enrichment in the tested gene sets. In the remaining part of this article only genes that were identified by GSEA as contributing to the significant enrichment in the identified significant gene sets are referred to as significant genes.

The significantly affected genes by model steatogenic drugs were uploaded to the Database for Annotation, Visualization, and Integrated Discovery (DAVID) Bioinformatics Resource, where the Functional Annotation Clustering tool generated clusters of overrepresented Gene Ontology (GO) terms [31], [32]. The Mouse Genome, 430 2 PM, was used as a background for the GO analysis of the mouse PCLS. After correction for false discovery rate (FDR) ≤0.005 (Benjamini Hochberg), the GO terms were selected for further analysis and interpretation.

In addition, we applied another open access data mining tool- Search Tool for the Retrieval of Interacting Genes/Proteins 8.2 (STRING) to perform gene functional clustering, which was visualized as networks. STRING constructs these networks using information from known and predicted protein-protein and gene-gene interactions present in curated as well as experimental databases, using statistical algorithms [33]. To construct gene functional networks in STRING, significant genes identified by GSEA in gene sets related to energy metabolism (i.e. glucose metabolism, lipid metabolism, fatty liver, peroxisomes, mitochondrial diseases, and drug metabolism) were used as input to construct gene functional networks.

### Biomarker Identification

To identify biomarkers specific for the steatogenic drugs, the significant genes found by GSEA in gene sets related to energy metabolism (i.e. gene sets called glucose metabolism, lipid metabolism, fatty liver, peroxisomes, mitochondrial diseases, and drug metabolism) were analysed by Venn diagrams using an open access online tool http://bioinfogp.cnb.csic.es/tools/venny/index.html. Genes, which were upregulated (FC≥1.5) in PCLS by AMI and VA, were selected as candidate biomarkers for PPARs agonists. Genes, which were uniquely downregulated by TET (FC≥−1.5), were selected as potential biomarkers for TET-like acting compounds. Subsequently, expression of the selected genes, derived from the normalized DNA microarray data, were log2 transformed, median centered, subjected to hierarchical clustering analysis (HCA), and was presented as heat maps using default options in Genesis (http://genome.tugraz.at/genesisserver/genesisserver_description.shtml). To confirm the specificity of the identified genes as candidate biomarkers for the steatogenic compounds, their expression was tested in data obtained from PCLS exposed to different classes of hepatotoxicants i.e. cholestatic and necrotic compounds. The gene expression found in PCLS exposed to the cholestatic and the necrotic drugs were processed as described above for the steatogenic drugs.

### PPAR Gene Reporter Assays

PPARα, PPARγ and PPARβ/δ CALUX cell lines were obtained from BioDetection Systems B.V. (BDS, Amsterdam, The Netherlands). These are based on human U2-OS cells (American Collection Cell Culture (ATCC), stably transfected with the relevant human PPAR expression plasmid and a luciferase reporter construct [34], [35] Man et al., unpublished). All lines were cultured as described before in a 1∶1 mixture of Dulbecco’s modified Eagle’s medium and Ham’s F12 medium (DF), (Invitrogen, Breda, the Netherlands) supplemented with 7.5% fetal bovine serum (FBS), 1% nonessential amino acids and (pen/strep) (Invitrogen) [35], [36]. Once a week, 200 µg/mL G418-disulfate was added to the culture medium as a selection pressure to maintain cells containing the construct. PPAR CALUX was assayed as before [35], [36]. For this, 200 µL of cell suspension in phenol-free DF supplemented with 5% dextran coated charcoal-stripped FBS was added to each well of 96-well plates. Test compounds were added to the culture medium after 24 h. Positive controls were known agonists of PPARα, PPARγ and PPARβ/δ, i.e. GW7647, rosiglitazone, and L165,041, respectively. Antagonistic activity was tested additionally in the presence of EC50 levels of agonist, i.e. 3e–9M, 1e–7M, and 8e–8M for GW7647, L165,041, and rosiglitazone respectively, essentially as before [35], [36]. The threshold for antagonism was set at 10% repression of agonist activity, in the absence of cytotoxicity. The medium was removed after 24 h, and the cells were washed and lysed before luciferase reagent was added and its activity was measured. For each test compound at last 2 independent experiments were carried out in triplicate. Luciferase activity per well was measured as relative light units. Fold induction was calculated by dividing the mean value of light units from exposed and non-exposed (solvent control) wells by plotting them in Excel.

## Results

### Slices Viability

To select one non-cytotoxic drug concentration for the gene expression profiling studies, PCLS were incubated for 24 h with model steatogenic compounds (AMI, VA, or TET) at different concentrations or the corresponding vehicle (control). Viability of PCLS treated with the drugs was assessed by ATP content normalized on protein level and compared to control incubations. There was no dose dependent drug-induced decrease in viability compared to controls by any of the tested drugs (Figure 1A–C). For the gene expression profiling studies, 50, 200 and 40 µM for AMI, VA and TET were applied, respectively, because these concentrations were non-toxic and similar concentrations had been used in other studies [37]–[39].

### Transcriptome Data Analysis

**Figure 2:**
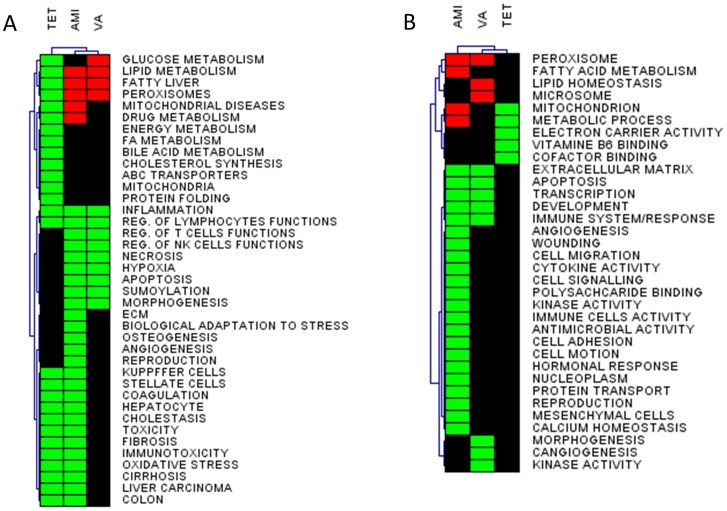
Effects of steatogenic drugs on gene expression in mouse PCLS. **A.** PCLS obtained from 5 mice were treated with 50 µM amiodarone (AMI), 200 µM of valproic acid (VA), 40 µM of tetracycline (TET) or vehicle for 24 h and subjected to Affymetrix microarray analysis. The biological processes in the heat map correspond to gene sets significantly affected according to GSEA (p<0.05, FDR<0.05). Processes that were upregulated are represented by red colour, the downregulated processes are depicted in green, and unaffected processes in black. **B.** Gene Ontology (GO) analysis of the significant genes identified by GSEA (p<0.05, FDR<0.05) was performed in DAVID. GO terms were considered to be significant if p<0.005, FDR<0.005. The significant GO terms were grouped into GO annotation clusters and are depicted as a heat map. For explanation of the colours see Figure 2A.

To study effect of AMI, VA, and TET on global gene expression in PCLS, DNA microarray analysis was performed. The array data were analysed by open access and commercial bioinformatics tools. Primarily, our interpretation focussed on assessing effects of steatogenic compounds on general biological processes, for which gene sets were generated using the literature data-mining tool, ANNI. In GSEA, we tested in total 47 gene sets related to general biological processes (e.g. inflammation, sumoylation, protein folding), hepatic functions (e.g. bile acid metabolism, cholesterol synthesis, lipid metabolism), and functions unrelated to liver (e.g. osteogenesis, kidney, brain, heart) (Table S1). The latter gene sets were included as a negative control. All significantly altered gene sets were subjected to HCA and are depicted as a heat map (Figure 2A); unaffected gene sets, including i.a. kidney, brain and heart, are not shown. Gene sets affected by AMI and VA shared the most similarities and clustered together. While gene sets affected by TET clustered apart from AMI and VA (Figure 2A). The most remarkable differences in AMI- and VA- versus TET-treated samples were found in gene sets related to lipid metabolism, fatty liver, and peroxisomes, which were upregulated by both AMI and VA, and downregulated by TET. Moreover, AMI and VA treatments downregulated gene sets related to necrosis, hypoxia, sumoylation, and regulation of T- and NK- cells functions, while these gene sets were unaffected by TET. Moreover, only TET-treatment downregulated gene sets related to other hepatic functions including bile acid metabolism, FA metabolism, ABC transporters, and cholesterol synthesis (Figure 2A).

Next, we extracted the significant genes altered in the gene sets identified by GSEA (p<0.05, FDR<0.05). Within the significantly enriched gene sets, a total of 774 genes were identified for AMI (93 upregulated and 681 downregulated), 348 genes for VA (45 upregulated and 303 downregulated), and 492 genes for TET (all downregulated). These genes were uploaded to DAVID for identification of GO terms. The GO analysis showed a total of 274 (24 upregulated and 250 downregulated), 152 (18 upregulated and 136 downregulated), and 18 (downregulated) GO terms for AMI, VA, and TET, respectively (p<0.005, FDR<0.005) (Tables S2A–C). The identified GO processes were grouped into GO annotation clusters, which were further analysed by HCA and are presented as a heat map (Figure 2B). In general, AMI and VA upregulated GO annotation clusters related to lipid metabolism and organelles involved in this process (e.g. mitochondrion (AMI) and peroxisomes (AMI and VA)). Additionally, AMI and VA downregulated several GO annotation clusters affiliated to immune functions, extracellular matrix, and development (Figure 2B). TET downregulated GO clusters related to mitochondrion and processes localized in this organelle, such as electron carrier activity (Figure 2B).

A similar type of analysis, using all the significant genes and individual GO terms, was performed by means of Venn diagrams. These results were in line with GSEA and GO annotation clusters analysis and showed that the biggest overlap was for AMI and VA, followed by a lower number of similarities between AMI and TET, and the least similarities were observed between VA and TET (Figure S3 A–B).

**Figure 3:**
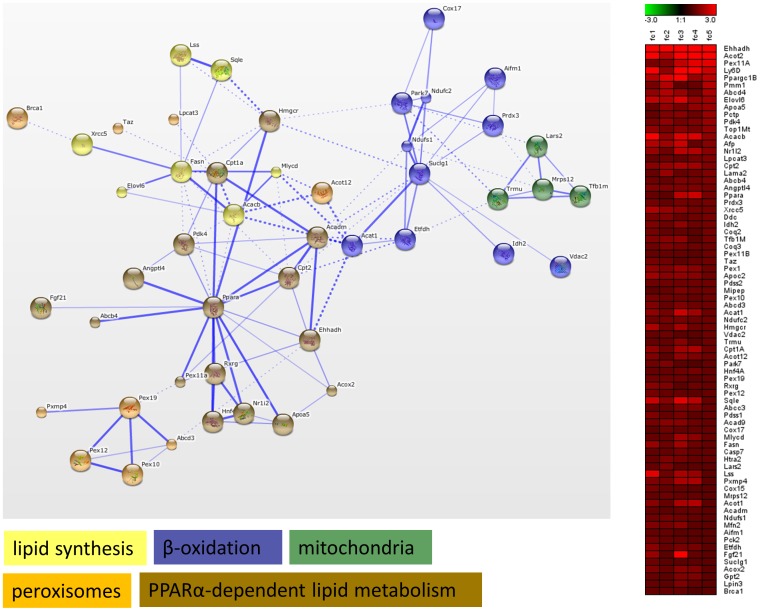
Functional clustering of genes involved in energy metabolism (amiodarone). Genes related to energy metabolism identified by GSEA as being significantly altered upon amiodarone (AMI) treatment were subjected to functional clustering in STRING. Functional clusters such as lipid synthesis, β-oxidation, mitochondria, peroxisomes, and PPARα -dependent lipid metabolism were identified. Information about fold change (FC = treatment vs. control) for the analysed genes in individual mice is presented as a heat map. Genes that did not form connected nodes were removed from the presented clusters. Thicker lines represent stronger associations between genes. Inter-cluster edges are represented by dashed-lines. The bigger spheres represent genes coding for proteins with known structure. Smaller spheres represent genes coding proteins for which no structural information is available.

**Figure 4:**
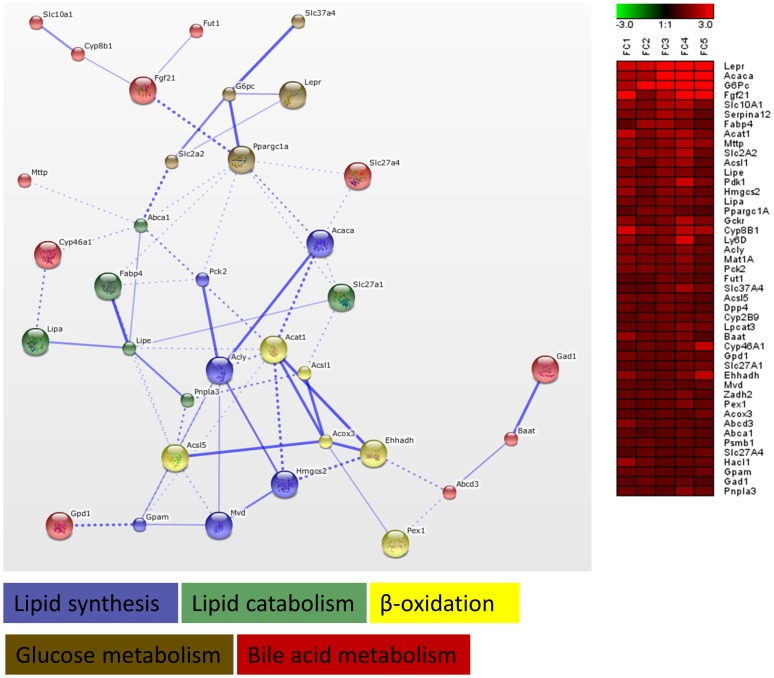
Functional clustering of genes involved in energy metabolism (valproic acid). Genes related to energy metabolism identified by GSEA as being significantly altered upon valproic acid (VA) treatment were subjected to functional clustering in STRING. Functional clusters such as lipid synthesis, lipid catabolism, β-oxidation, glucose metabolism, and bile acid metabolism have been identified. Information about fold change (FC = treatment vs. control) for the analysed genes in individual mice is presented as a heat map. For further explanation of the networks see Fig. 3.

**Figure 5:**
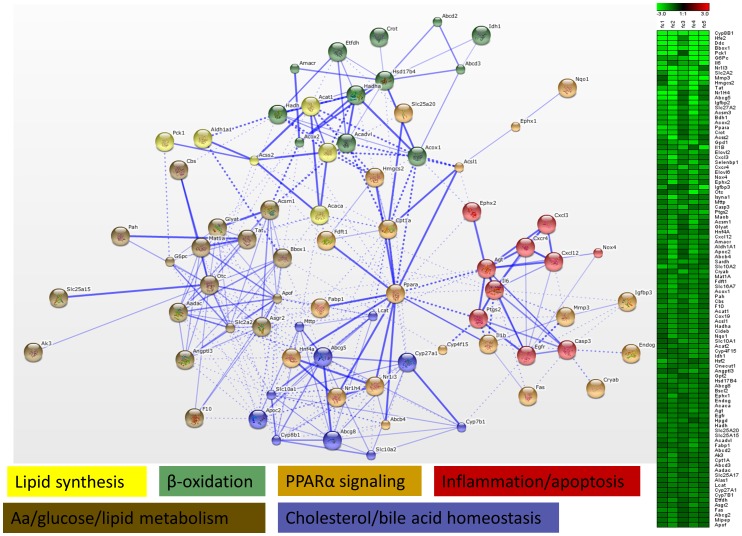
Functional clustering of genes involved in energy metabolism (tetracycline). Genes related to energy metabolism identified by GSEA as being significantly altered upon tetracycline (TET) treatment were subjected to functional clustering in STRING. Functional clusters such as lipid synthesis, β-oxidation, PPARα signaling, inflammation/apoptosis, amino acids (aa)/glucose/lipid metabolism, and cholesterol/bile acid homeostasis were identified. Information about fold change (FC = treatment vs. control) for the analysed genes in individual mice is presented as a heat map. For explanation of the networks see Fig. 3.

In a further analysis, we focused on significant genes identified by GSEA in gene sets related to energy metabolism (p<0.05, FDR<0.05). To this end, for each of the 3 treatments, significant genes were extracted from selected gene sets, i.e. glucose metabolism, lipid metabolism, fatty liver, peroxisomes, mitochondrial diseases and drug metabolism. These genes were uploaded to STRING for functional clustering, which resulted in the generation of distinct networks for each drug. However, both AMI and VA upregulated several genes, which grouped into processes related to lipid metabolism. AMI upregulated gene functional clusters related to *Pparα*-dependent lipid metabolism, β-oxidation, peroxisomes, mitochondria and lipid synthesis (Figure 3). The network generated for VA contained gene clusters such as lipid synthesis, lipid catabolism, β-oxidation, glucose metabolism and bile acid metabolism (Figure 4). In contrast to AMI and VA, TET downregulated functional clusters related to lipid synthesis, β-oxidation, *Pparα* signaling, inflammation, apoptosis, and other clusters related to energy and bile acid homeostasis (Figure 5). Remarkably, these networks included several *Pparα* target genes, such as *Cpt1a* (AMI & TET), *Mttp*, *Fabp*, *Acat1* (TET&VA), *Fgf21* (AMI &VA), as well as processes that are governed by *Pparα* (e.g. β-oxidation, lipid, and bile acid metabolism). Based on these observations, we speculated that both AMI and VA act as *Pparα* selective agonists, while TET could be an antagonist of *Pparα*.

### PPAR α, (β/δ), and γ Gene Reporter Assays

**Figure 6:**
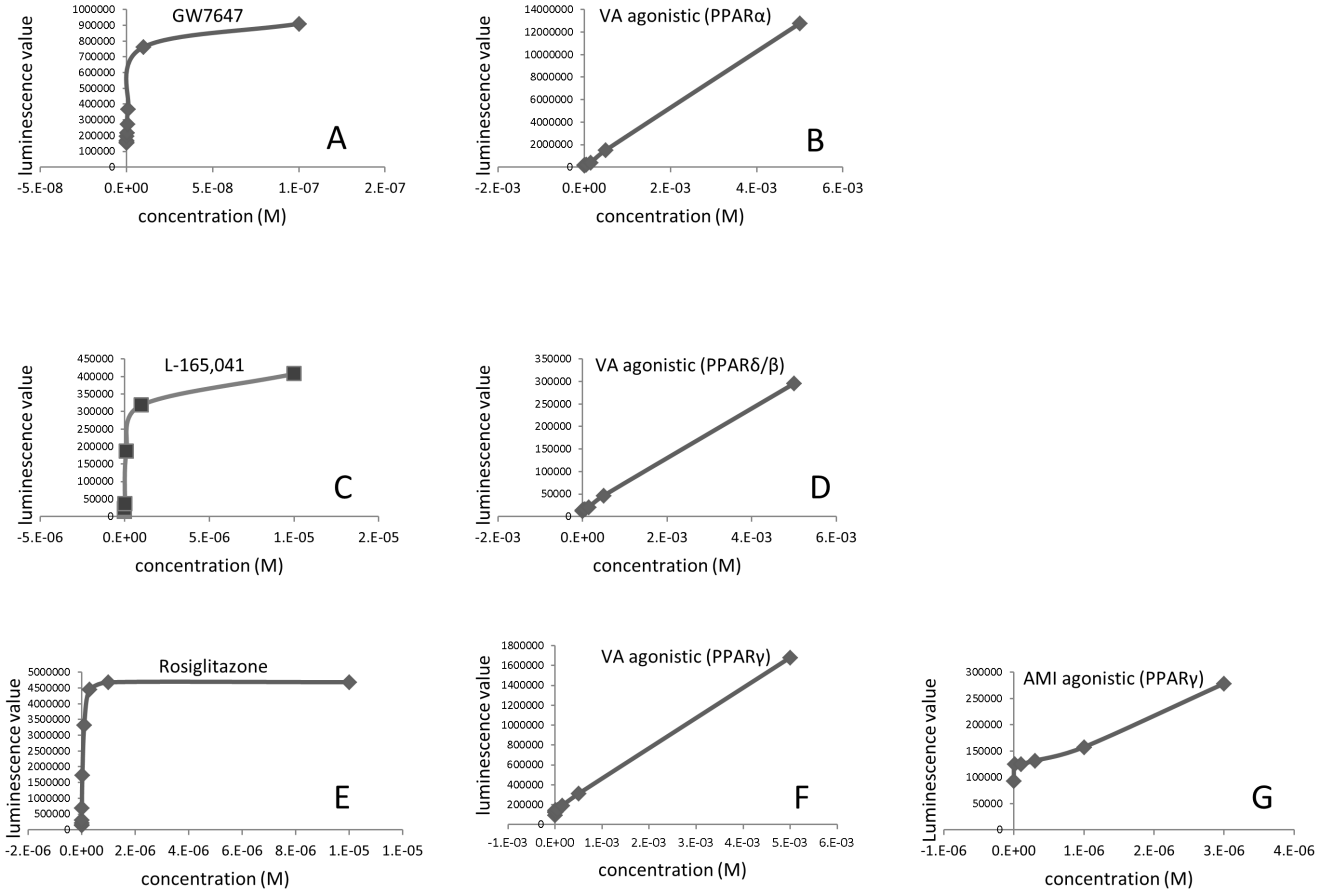
Effect of valproic acid and amiodarone on PPARα, PPAR β/δ, and PPARγ gene reporter assays. Luciferase activity of PPARα CALUX cells upon exposure to PPARα agonists: GW7647 (A) and valproic acid (B). Luciferase activity of PPAR β/δ CALUX cells upon exposure to PPAR β/δ agonists: L-165, 041 (C), and valproic acid (D). Luciferase activity of PPAR**γ** CALUX cells upon exposure to PPAR**γ** agonists**:** rosiglitazone (E), valproic acid (F), and amiodarone (G). Data are corrected for solvent control values and expressed as means±standard errors (n = 3). X axis represents concentration of the compounds [M] and y axis represents luciferase units. AMI stands for amiodarone, VA-valproic acid, and TET-tetracycline.

To assess whether the tested compounds act as PPARα agonists or antagonist, we used a human stable PPARα gene reporter assay, and also tested the same compounds in human PPAR β/δ and human PPAR γ reporter assays to verify any possible coactivity towards other members of closely related PPAR family. The assays showed that VA is an agonist of PPAR α, β/δ, and γ (Figure 6B, D, F). AMI was an agonist of PPARγ (Figure 6G). TET acted neither as an antagonist or agonist of any of the tested PPARs (Figure S4).

### Identification of Biomarkers

**Figure 7:**
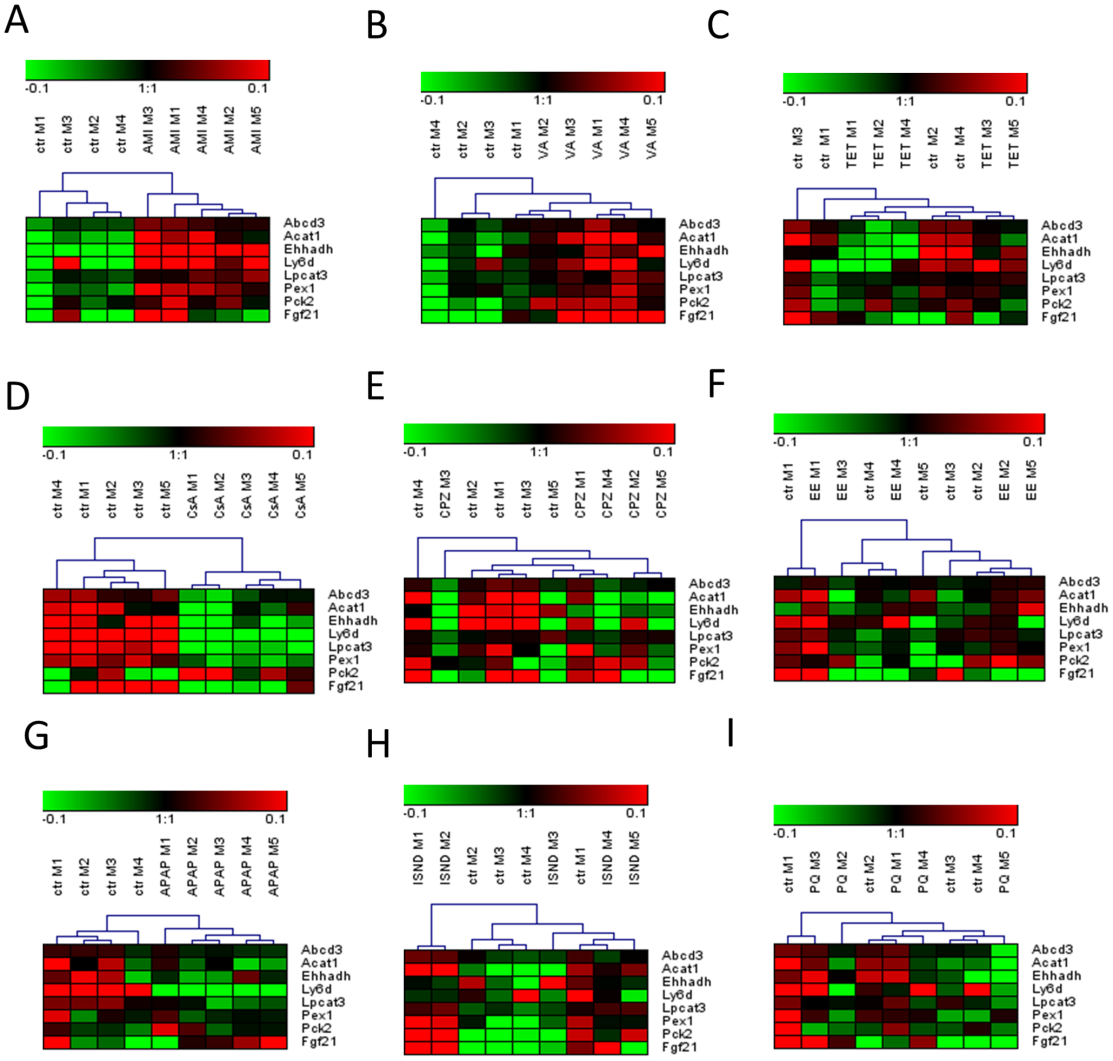
Identification of potential biomarkers for PPAR agonists in mouse PCLS. PCLS obtained from 4 or 5(amiodarone (A), valproic acid (B), or tetracycline(C)), cholestasis (cyclosporin A (D), chlorpromazine (E), or ethinyl estradiol (F)), necrosis (acetaminophen (G), isoniazid (H), or paraquat (I)), or controls. GSEA led to the identification of 8 genes upregulated by amiodarone and valproic acid, which were considered as candidate biomarkers for PPAR agonists. mRNA expression values for the selected biomarkers are derived from DNA-microarrays and results are presented as heat maps of log2, median centered gene expression values subjected to HCA. Red and green indicate expression higher and lower, respectively, than the average expression of all samples within the same heat map. AMI stands for amiodarone, VA-valproic acid, TET- tetracycline, CsA-cyclosporin A, CPZ- chlorpromazine, EE- ethinyl estradiol, APAP-acetaminophen, ISND- isoniazid, PQ- paraquat, and ctr- controls, M1 represents PCLS obtained from liver of mouse nr 1 etc.

**Figure 8:**
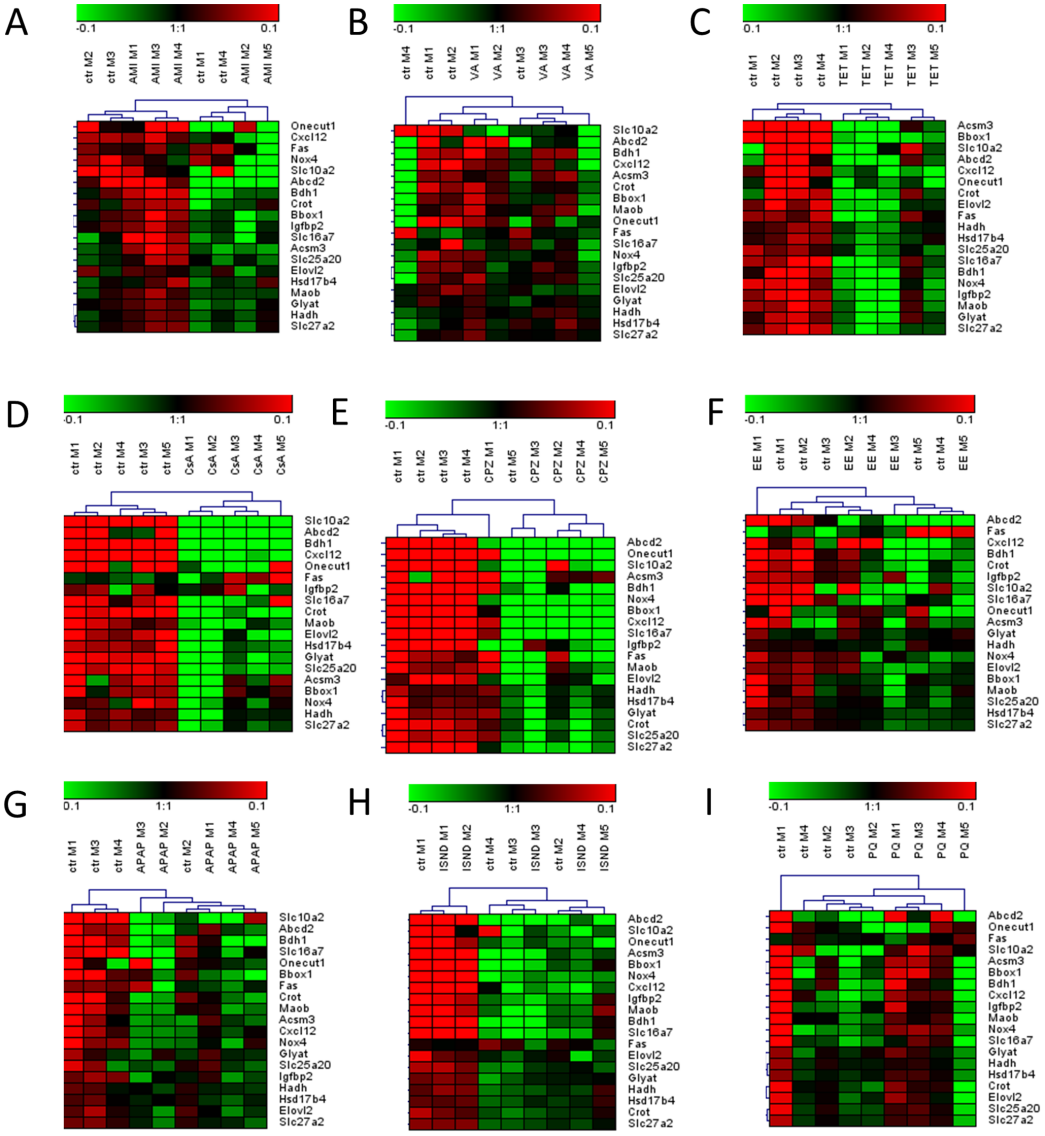
Identification of potential biomarkers for tetracycline-like acting compounds in mouse PCLS. PCLS obtained from 4 or 5 mice were exposed for 24(amiodarone (A), valproic acid (B), or tetracycline (C)), cholestasis (cyclosporin A (D), chlorpromazine (E), or ethinyl estradiol (F)), necrosis (acetaminophen (G), isoniazid (H), or paraquat(I)), or controls. GSEA led to the identification of 19 genes downregulated by tetracycline (TET) treatment, which were considered as candidate biomarkers for TET-like acting compounds. mRNA expression values for the selected biomarkers are derived from DNA-microarrays, and results are presented as heat maps of log2, median centered gene expression values subjected to HCA. For explanation of the colours and abbreviations see Figure 7.

To identify candidate biomarkers for steatogenic drugs, we focused on significant genes related to energy metabolism extracted from the selected gene sets identified by GSEA, i.e. glucose metabolism, lipid metabolism, fatty liver, peroxisomes, mitochondrial diseases and drug metabolism (Figure 2). We tried to distinguish between steatogenic compounds directly interfering with PPARs, such as AMI and VA, and compounds acting like TET. Therefore, as candidate biomarkers for PPAR agonists, we tested 8 genes upregulated by both AMI and VA (Figure S5). Although 2 out of the tested 8 genes were altered by all the studied compounds, only AMI and VA upregulated these 2 genes, while TET treatment led to their downregulation (*Abcd3, Acat1*). The 8 candidate biomarkers for PPAR agonists were subjected to HCA, which led to a good separation between treatment and control for AMI and VA (Figure 7 A–B). With respect to the identification of candidate biomarkers for drugs acting similarly to TET, we selected 77genes exclusively downregulated by this drug (Figure S5). HCA was carried out for these 77 genes and eventually 19 genes leading to the best resolution between control and TET-treated slices were selected (Figure 8C). More detailed information on the functions of the candidate biomarkers is given in Table S3.

In order to check specificity of the selected genes to screen for PPAR agonists or TET-like acting compounds, their expression was analysed in slices treated with different classes of hepatotoxicants, such as model cholestatic compounds (CsA, CPZ, and EE) (Figures 7 D-F and 8 D–F), and model necrotic compounds (APAP, ISND, and PQ) (Figures 7G-I and 8 G–I). With regard to candidate biomarkers for drugs interfering with PPARs, we did not detect a similar pattern of expression for any of the analysed hepatotoxicants (Figure 7 D–I). With respect to candidate biomarkers for TET-like drugs, only 2 of the tested hepatotoxicants, i.e. CsA and CPZ, gave a similar gene expression pattern as TET (Figure 8 D–E).

### Comparative Data Analysis: Relevance for Mouse in vivo and Human Primary Hepatocytes

**Figure 9:**
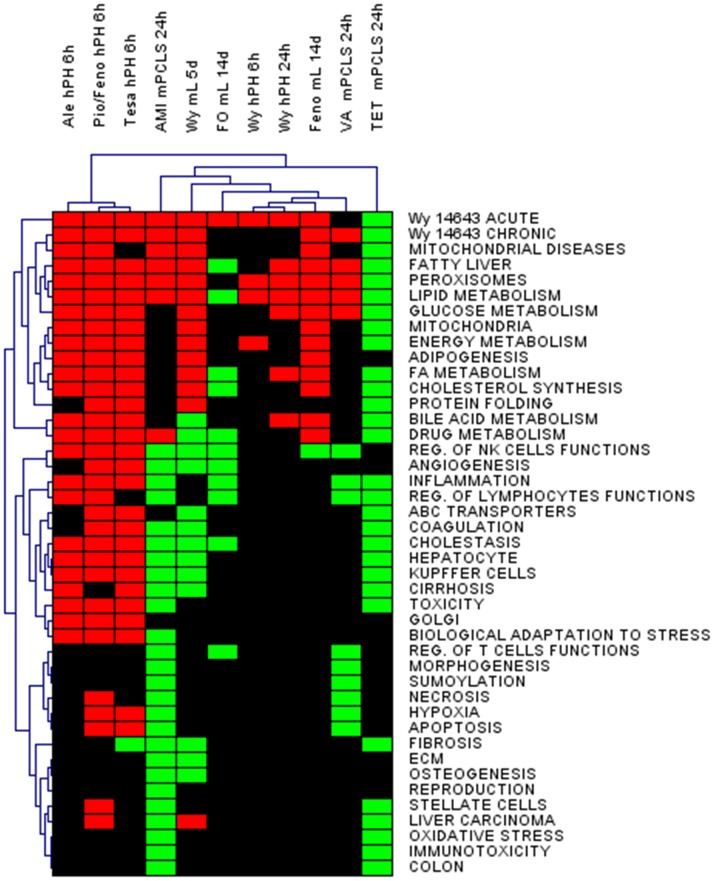
Comparative data analysis: relevance for mouse in vivo and human primary hepatocytes. Publically available transcriptomics data (Gene Expression Omnibus) relevant for the actions of known PPAR agonists in mouse liver *in vivo* and human primary hepatocytes were used. The heat map represents significant gene sets (GSEA p<0.05, FDR<0.05), which were subjected to HCA. Gene sets were obtained using the ANNI text mining tool. Processes that were upregulated are represented by red colour, the downregulated processes are depicted in green, and unaffected processes are in black. Ale stands for aleglitazar (double PPARα/γ agonist), Pio/Feno-pioglitazone/fenofibrate (PPAR γ/PPARα agonists), Tesa-Tesaglitazar (double PPAR γ/α agonist), AMI-amiodarone (PPAR γ agonist), VA-valproic acid (triple PPARα/(β/δ)/γ agonist), TET-tetracycline, Wy-Wy14643, FO-fish oil, m-mouse, h-human, PCLS-precision cut liver slices, PH-primary hepatocytes, L- liver in vivo.

To validate that mouse PCLS can be used as an alternative to animal testing and it is a relevant model for the human situation to study actions of model steatogenic compounds, another analysis using publically available transcriptomics data was performed. The data were derived from mouse livers and human primary hepatocytes exposed to known PPAR agonists. In the *in vivo* experiments, mice were treated with different PPARα agonists, such as Wy14643 (for 5 days), fenofibrate and fish oil (for 14 days). Primary human hepatocytes were exposed to Wy14643 for 6 and 24 h, as well as to double agonists of PPARα and PPARγ, such as aleglitazar, pioglitazone/fenofibrate, and tesaglitazar for 6 h. GSEA showed that in all models, the known PPAR agonists upregulated gene sets related to energy metabolism as well as chronic and/or acute actions of Wy14643. Moreover, compounds identified as PPAR agonists (AMI and VA) gave a similar results as known PPAR agonists in gene sets related to lipid and energy metabolism (Figure 9). In addition to the commonly regulated gene sets by PPAR agonists, each of the analysed PPAR ligands uniquely regulated other gene sets related to i.a. immunity or morphogenesis. With regard to TET, next to mentioned above downregulation of gene sets related to energy metabolism, it also downregulated gene sets related to actions of Wy14643 (Figure 9).

## Discussion

In this study we used mouse PCLS as an *in vitro* model to study the mechanism of action of model steatogenic compounds. We applied a toxicogenomics approach in combination with gene reporter assays to examine the value of mouse PCLS as an alternative to animal testing and relevant model for humans.

Effects on ATP content was used for dose selection since this is a generally accepted assay for assessing slice viability. Based on the ATP levels, none of the concentrations of the steatogenic drugs affected viability in comparison to the controls (Figure 1). For the gene expression profiling studies, 50, 200, and 40 µM for AMI, VA, and TET were used similarly as in other studies [37]–[39].

Transcriptome data analysis including GSEA, GO analysis, and functional clustering, showed that AMI and VA acted similarly, indicating that these 2 drugs share some mechanisms of action. GSEA and GO analysis identified that both drugs upregulated processes related to lipid metabolism, and downregulated gene sets and GO-terms related to diverse immune processes (Figure 2 A–B, Figure S3, and Tables S2A–B). An additional gene functional cluster analysis in STRING, showed that both AMI and VA upregulated several clusters related to lipid homeostasis, such as lipid synthesis or β-oxidation. These clusters contained known PPARα target genes, exemplified by *Pex11a*, *Elovl6*, *Pdk4*, *Cpt1a* and *Cpt2* (upregulated by AMI) and *Acsl1*, *Acox3*, *Mttp* or *Fabp4* (upregulated by VA treatment), Figures 3–4. These results agree with the findings of others; in mouse liver AMI upregulated *Acox2*, *Cpt1*, *Cpt2* and *Mttp,* indicative of activation of lipid catabolism via PPARα [16]. Gene expression profiles generated by VA in rat hepatocytes and livers were highly similar to gene expression profiles obtained with known PPARα agonists, thereby classifying VA as a PPARα agonist [19]. However, our findings contrast to those of others who found that AMI and VA toxicity is related to the impairment of fatty acid β-oxidation after chronic treatment [14], [40], [41]. This discrepancy may be caused by the different duration used in these studies. AMI displays dual effects on mitochondrial respiration characterized by an initial increased rate of β-oxidation followed by a marked inhibition [42]. The duration of 24 h we used may be too short to induce toxicity seen with chronic exposure to AMI and VA *in vivo* [14], [40], [41]. Toxicity in chronic exposure to AMI was caused by accumulation of its toxic metabolites inhibiting mitochondrial proteins [42], [43]. Similarly, biotransformation of VA results in formation of 50 metabolites that inhibit several enzymes of mitochondrial β-oxidation after chronic treatment [17].

To determine whether the upregulation of PPARα target genes was caused by direct binding of AMI and VA to PPARα, a PPARα gene reporter assay was used. We also applied PPAR β/δ and PPAR γ reporter assays, since several PPAR ligands are trans-activating multiple forms of PPARs [18]. Indeed, VA not only activated the PPARα reporter, but the β/δ, and γ assays, thereby acting as a triple PPARα/(β/δ)/γ agonist (Figure 6 B, D, and G). Activation of the PPARα reporter by VA agrees with findings that it induces a gene expression pattern comparable to PPARα agonists [19]. There seem to be no other reports on agonistic effects of VA on PPARβ/δ or PPARγ in the liver. Surprisingly, AMI turned out to be a PPARγ agonist instead of PPARα agonists, as speculated based on our transcriptomics data and the literature reports (Figure 6F) [16]. PPARγ is highly expressed in adipose tissue, where it controls adipogenesis and adipocyte functions [44]. Under physiological conditions, PPARγ has a low expression in the liver, but has an elevated expression in the steatotic liver [45]. Consistent with these notions, we observed that some PPARγ target genes involved in lipogenesis (*Elovl6* and *Fasn*) were upregulated by AMI. In addition, we observed upregulation of genes involved in mitochondrial and peroxisomal β-oxidation (*Cpt1a*, *Cpt2*, and *Pex11a*). These findings correspond with other reports in which human and rat primary hepatocytes treated with synthetic PPARγ agonists upregulated the same genes [46], [47].

We also found that AMI and VA downregulated several processes related to inflammation and extracellular matrix. These observations are in line with known anti-inflammatory and anti-fibrotic actions of PPAR agonists on non-parenchymal liver cells [18].

With regard to effects of TET on gene expression, GSEA and GO analysis showed that TET downregulated processes related to lipid metabolism (Figure 2 A–B). These results correspond with the known negative interference of TET with mitochondrial β-oxidation in rat liver associated with steatosis [8], [48]. We also found that TET downregulated expression of PPARα and its target genes (e.g. *Cpt1a*, *Fabp1*; Figure 5), which agrees with findings in the mouse liver [49]. However, it has been previously shown that TET upregulated expression of lipogenic genes, such as FASN, SREBP1C and PPARγ, in the human HepaRG liver cell line, which we did not find in mouse PCLS [37]. The explanation for this difference could be related to inter-species differences and/or the composition of both models. HepaRG consists of hepatocyte-like cells, while PCLS contain parenchymal and non-parenchymal cells, whose interactions are crucial for regulation of lipid metabolism [18]. Moreover, in the mouse liver, TET-induced steatosis has been associated with upregulation of fatty acids elongases (*Elovl* 3, 5, 6) without altered expression of other genes involved in *de novo* lipogenesis, such as *FasN*, *Srebp1c, and* Pparγ [9]. Therefore, steatogenic properties of TET seem to be species- and model- specific and the underlying mechanism requires additional studies. Furthermore, based on the outcome of our PPAR assays, it can be concluded that the downregulation of PPAR target genes is not related to direct antagonistic effects of TET on any of the PPARs tested (Figure S4).

Since the outcome of our research points towards PPAR agonistic activity of AMI and VA and negative regulation of lipid metabolism by TET, we propose 2 dedicated sets of biomarkers for PPAR agonists and TET-like acting compounds. Analysis of candidate biomarkers for PPARs agonistic activity showed that the selected genes were specifically upregulated by both AMI and VA and were not altered by other types of hepatotoxicants (Figure 7 A–I). With regard to biomarkers for TET-like acting drugs, the selected candidate genes separated TET-treated samples from control slices but they did not separate slices treated with AMI, VA, EE, as well as necrotic compounds. Remarkably, the biomarker genes for TET were also downregulated by CsA and CPZ (Figure 8 D–E). This indicates that TET shares mechanism of action with CsA and CPZ, which both negatively affect mitochondrial activity by induction of oxidative stress [50], [51]. Although CsA and CPZ have been regarded here as model cholestatic compounds, they can also cause steatosis, likely by impairment of mitochondrial functions [52]–[55].

Comparative analysis of gene expression patterns in mouse PCLS exposed to steatogenic drugs versus human primary hepatocytes and livers of mice exposed to known PPAR agonists clearly showed similarities in regulation of gene sets related to lipid metabolism and PPAR signaling. This supports the use of mouse PCLS as an alternative to animal testing and human *in vitro* models for the identification of early mechanisms involved in drug-induced perturbations in lipid homeostasis (Figure 9).

In summary, mouse PCLS in combination with transcriptomics, can be used to study early mechanisms of action induced by model steatogenic drugs. Both AMI and VA affect processes related to lipid metabolism by binding to master regulators of lipid homeostasis, i.e. PPARγ and PPARα/(β/δ)/γ respectively and regulating expression of their target genes. TET downregulated processes related to mitochondrial functions and lipid metabolism. Regarding the comparative GSEA analysis, the results obtained in mouse PCLS are alike with mouse *in vivo* and human *in vitro* data, supporting mouse PCLS as a good alternative to animal testing and a valid model to study effects of steatogenic compounds in relation to the human situation. Two sets of the identified candidate biomarkers could be used to screen for compounds that alter lipid metabolism and as such may be hepatotoxic.

## Supporting Information

Figure S1 **Dose selection experiments for cholestatic and necrotic drugs.** Liver slices were incubated for 24 h with a range of concentrations for model cholestatic compounds: cyclosporin A (CsA) 1–100 µM, chlorpromazine (CPZ) 2–80 µM, ethinyl estradiol (EE) 0.1–100 µM, and model necrotic compounds: acetaminophen (APAP) 0.3–3 mM, isoniazid (ISND) 0.1–1 mM, paraquat (PQ) 1–10 µM, or corresponding controls. ATP content (nmol/mg of protein) was measured to assess liver slice viability. Each point is the mean±SD of 2 independent experiments (liver slices were isolated from livers of 2 mice) and each measurement was done in duplicate.
(PPTX)

Figure S2 **Viability of mouse liver slices upon treatment with cholestatic and necrotic drugs.** Liver slices were incubated for 24 h with pre-selected concentrations of model cholestatic compounds: cyclosporin A (CsA) 40 µM, chlorpromazine (CPZ) 20 µM, ethinyl estratiol (EE) 10 µM, and model necrotic compounds: acetaminophen (APAP), isoniazid (ISND), paraquat (PQ), or corresponding controls (ctr). ATP content (nmol/mg of protein) was measured to assess liver slice viability. Each point is the mean±SD of 5 independent experiments (liver slices were isolated from livers of 5 mice) and each measurement was done in duplicate. Slices viability was not significantly affected by any of the tested drug concentrations compared to control.
(PPTX)

Figure S3 **Comparative analysis of significant genes and GO processes affected by steatogenic drugs in mouse PCLS.** (A) Genes identified by GSEA as being significantly altered in PCLS upon amiodarone (AMI), valproic acid (VA), and tetracycline (TET) are shown as Venn diagrams. (B) The same genes were used for Gene Ontology (GO) analysis in DAVID and the significant GO terms (p<0.05, FDR<0.005) are shown in Venn diagrams.
(PPTX)

Figure S4 **Effect of tetracycline on PPARα-, PPAR β/δ-, and PPARγ gene reporter assays.** Luciferase activity of PPARα-, PPAR β/δ-, and PPAR**γ-** CALUX cells on exposure to corresponding agonists GW7647 (A), L-165, 041 (D), and rosiglitazone (G) respectively. Tetracycline (TET) was tested in both agonistic (B, E, H) and antagonistic (C, F, I) modes in the 3 PPAR-CALUX assays. Data are corrected for solvent control values and are expressed as means±standard errors (n = 3). X axis represents concentration of the tested compounds [M] and y axis represents luciferase units.
(PPTX)

Figure S5 **Identification of candidate biomarkers for steatogenic drugs in mouse PCLS.** To identify candidate biomarkers for steatogenic drugs, significant genes found by GSEA in precision cut liver slices (PCLS) treated with amiodarone (AMI), valproic acid (VA), and tetracycline (TET) were analysed by Venn diagrams. Eight overlapping, upregulated genes in PCLS treated with AMI and VA were considered as candidate biomarkers for PPARs agonists. Genes uniquely downregulated by TET (i.e. 77), were considered as candidate biomarkers for TET-like acting compounds.
(PPTX)

Table S1 **ANNI gene sets.** Gene sets related to diverse hepatic and non-hepatic functions were created in ANNI and were used in GSEA to detect major biological processes affected by treatment with amiodarone, valproic acid, and tetracycline.
(DOCX)

Table S2 **Total GO analysis.** Significant genes altered by steatogenic drugs in precision cut liver slices (PCLS) were subjected to Gene Ontology (GO) analysis in DAVID. The GO analysis identified 288 (27 up- and 261-down-regulated), 152 (18 up- and 136 down-regulated), and 21 (downregulated) GO terms for amiodarone, valproic acid, and tetracycline respectively.
(DOCX)

Table S3 **Candidate biomarkers.** Functions of candidate biomarkers for PPAR agonists and tetracycline (TET)-like acting compounds are derived from GeneCards http://www.genecards.org.
(DOCX)
