# Supplementary material for: Model Steatogenic Compounds (Amiodarone, Valproic Acid, and Tetracycline) Alter Lipid Metabolism by Different Mechanisms in Mouse Liver Slices
Source: PLoS One. 2014 Jan 29;9(1):e86795. doi: 10.1371/journal.pone.0086795 (PMC3906077; doi:10.1371/journal.pone.0086795)
Supplement: Figure S1 — Dose selection experiments for cholestatic and necrotic drugs. Liver slices were incubated for 24 h with a range of concentrations for model cholestatic compounds: cyclosporin A (CsA) 1–100 µM, chlorpromazine (CPZ) 2–80 µM, ethinyl estradiol (EE) 0.1–100 µM, and model necrotic compounds: acetaminophen (APAP) 0.3–3 mM, isoniazid (ISND) 0.1–1 mM, paraquat (PQ) 1–10 µM, or corresponding controls. ATP content (nmol/mg of protein) was measured to assess liver slice viability. Each point is the mean±SD of 2 independent experiments (liver slices were isolated from livers of 2 mice) and each measurement was done in duplicate. (PPTX) [file pone.0086795.s001.pptx]

## Slide 1
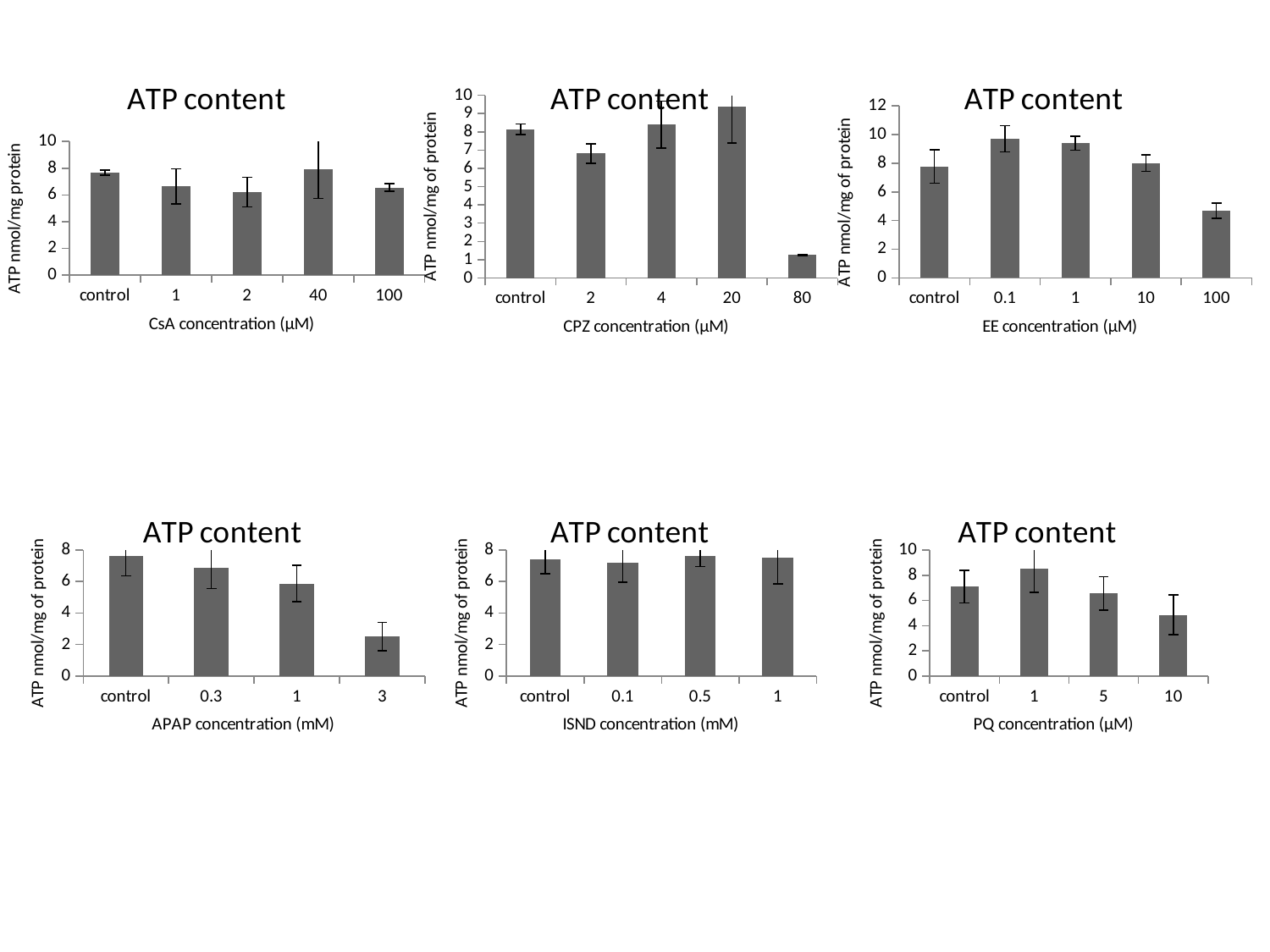

### Chart: ATP content
| Category | |
|---|---|
| control | 7.657835294117648 |
| 1 | 6.640200000000001 |
| 2 | 6.226200000000001 |
| 40 | 7.8902526315789485 |
| 100 | 6.552630000000001 |
### Chart: ATP content
| Category | |
|---|---|
| control | 8.1418575 |
| 2 | 6.8099625 |
| 4 | 8.406247500000001 |
| 20 | 9.380070000000002 |
| 80 | 1.261153125 |
### Chart: ATP content
| Category | |
|---|---|
| control | 7.765794448004201 |
| 0.1 | 9.701625857142856 |
| 1 | 9.393886025735293 |
| 10 | 8.009053362132352 |
| 100 | 4.700016746305419 |
### Chart: ATP content
| Category | |
|---|---|
| control | 7.602767200000001 |
| 0.3 | 6.885911199999999 |
| 1 | 5.8783144 |
| 3 | 2.4973815999999998 |
### Chart: ATP content
| Category | |
|---|---|
| control | 7.411930399999999 |
| 0.1 | 7.1833832 |
| 0.5 | 7.6079191999999995 |
| 1 | 7.5032216 |
### Chart: ATP content
| Category | |
|---|---|
| control | 7.090176800000001 |
| 1 | 8.5402625 |
| 5 | 6.5571128 |
| 10 | 4.8531952 |
