# Supplementary material for: Model Steatogenic Compounds (Amiodarone, Valproic Acid, and Tetracycline) Alter Lipid Metabolism by Different Mechanisms in Mouse Liver Slices
Source: PLoS One. 2014 Jan 29;9(1):e86795. doi: 10.1371/journal.pone.0086795 (PMC3906077; doi:10.1371/journal.pone.0086795)
Supplement: Figure S2 — Viability of mouse liver slices upon treatment with cholestatic and necrotic drugs. Liver slices were incubated for 24 h with pre-selected concentrations of model cholestatic compounds: cyclosporin A (CsA) 40 µM, chlorpromazine (CPZ) 20 µM, ethinyl estratiol (EE) 10 µM, and model necrotic compounds: acetaminophen (APAP), isoniazid (ISND), paraquat (PQ), or corresponding controls (ctr). ATP content (nmol/mg of protein) was measured to assess liver slice viability. Each point is the mean±SD of 5 independent experiments (liver slices were isolated from livers of 5 mice) and each measurement was done in duplicate. Slices viability was not significantly affected by any of the tested drug concentrations compared to control. (PPTX) [file pone.0086795.s002.pptx]

## Slide 1
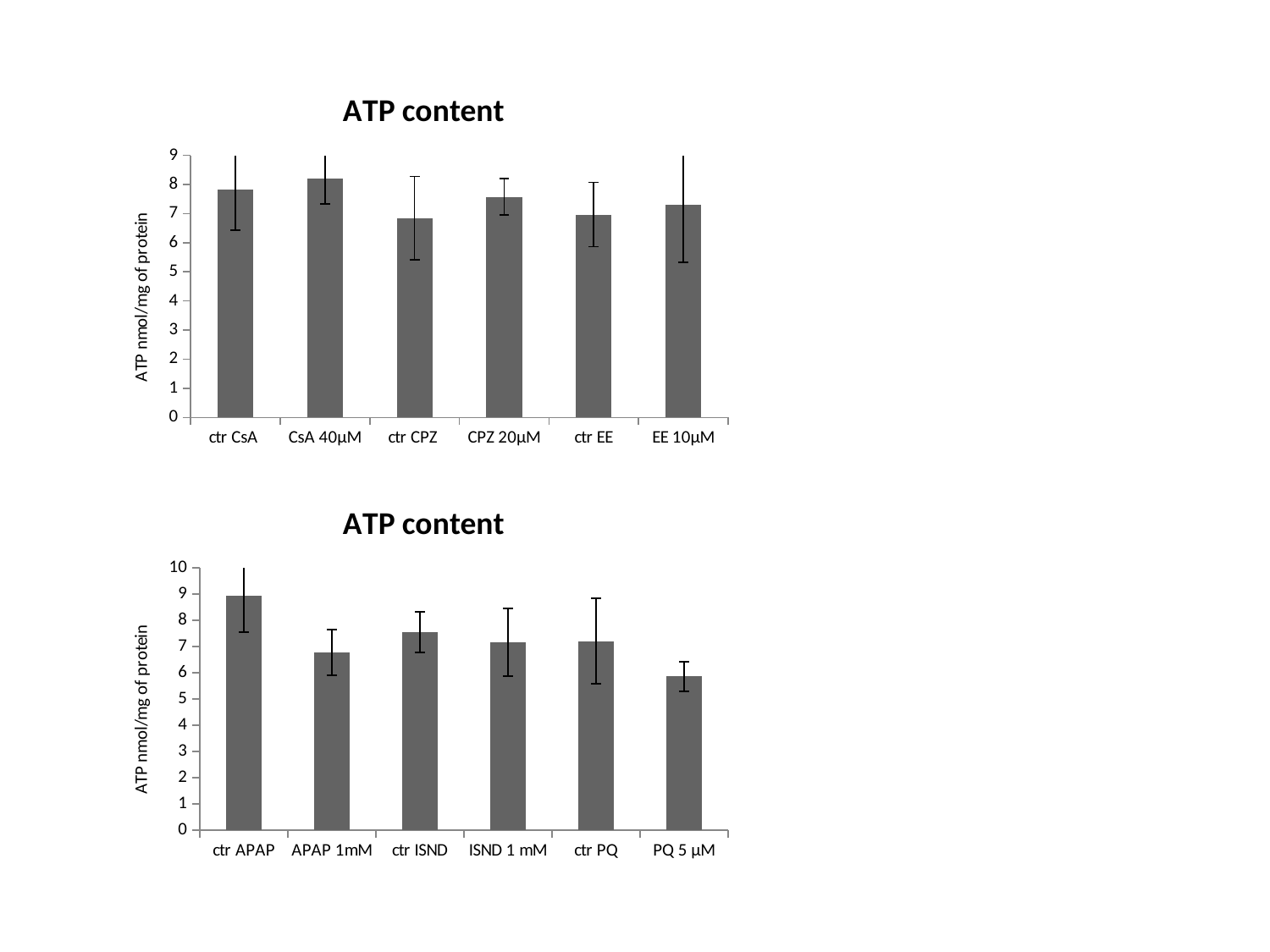

### Chart: ATP content
| Category | |
|---|---|
| ctr CsA | 7.832163583086729 |
| CsA 40µM | 8.19576178623126 |
| ctr CPZ | 6.848533724659702 |
| CPZ 20µM | 7.58067910346718 |
| ctr EE | 6.970625588947189 |
| EE 10µM | 7.294227989745822 |
### Chart: ATP content
| Category | |
|---|---|
| ctr APAP | 8.94432631429408 |
| APAP 1mM | 6.778414719738014 |
| ctr ISND | 7.556449417842678 |
| ISND 1 mM | 7.172468928279492 |
| ctr PQ | 7.210152221442748 |
| PQ 5 µM | 5.8567554948130525 |
