# Supplementary figures and images for: Model Steatogenic Compounds (Amiodarone, Valproic Acid, and Tetracycline) Alter Lipid Metabolism by Different Mechanisms in Mouse Liver Slices
Source: PLoS One. 2014 Jan 29;9(1):e86795. doi: 10.1371/journal.pone.0086795 (PMC3906077; doi:10.1371/journal.pone.0086795)

## Slide 1
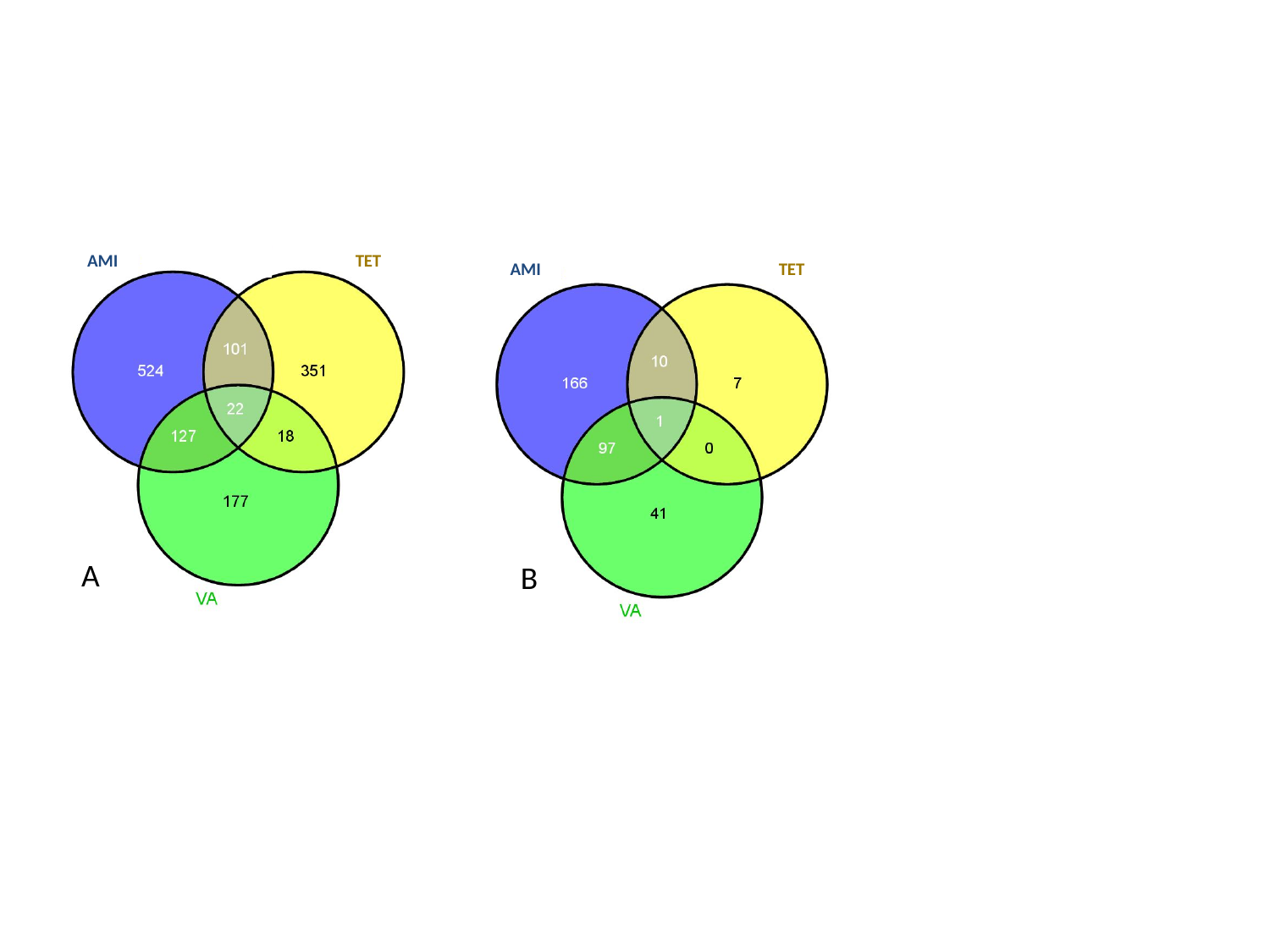

AMI
TET
AMI
TET
 A
B

Supplement: Figure S3 — Comparative analysis of significant genes and GO processes affected by steatogenic drugs in mouse PCLS. (A) Genes identified by GSEA as being significantly altered in PCLS upon amiodarone (AMI), valproic acid (VA), and tetracycline (TET) are shown as Venn diagrams. (B) The same genes were used for Gene Ontology (GO) analysis in DAVID and the significant GO terms (p<0.05, FDR<0.005) are shown in Venn diagrams. (PPTX) [file pone.0086795.s003.pptx]

## Slide 1
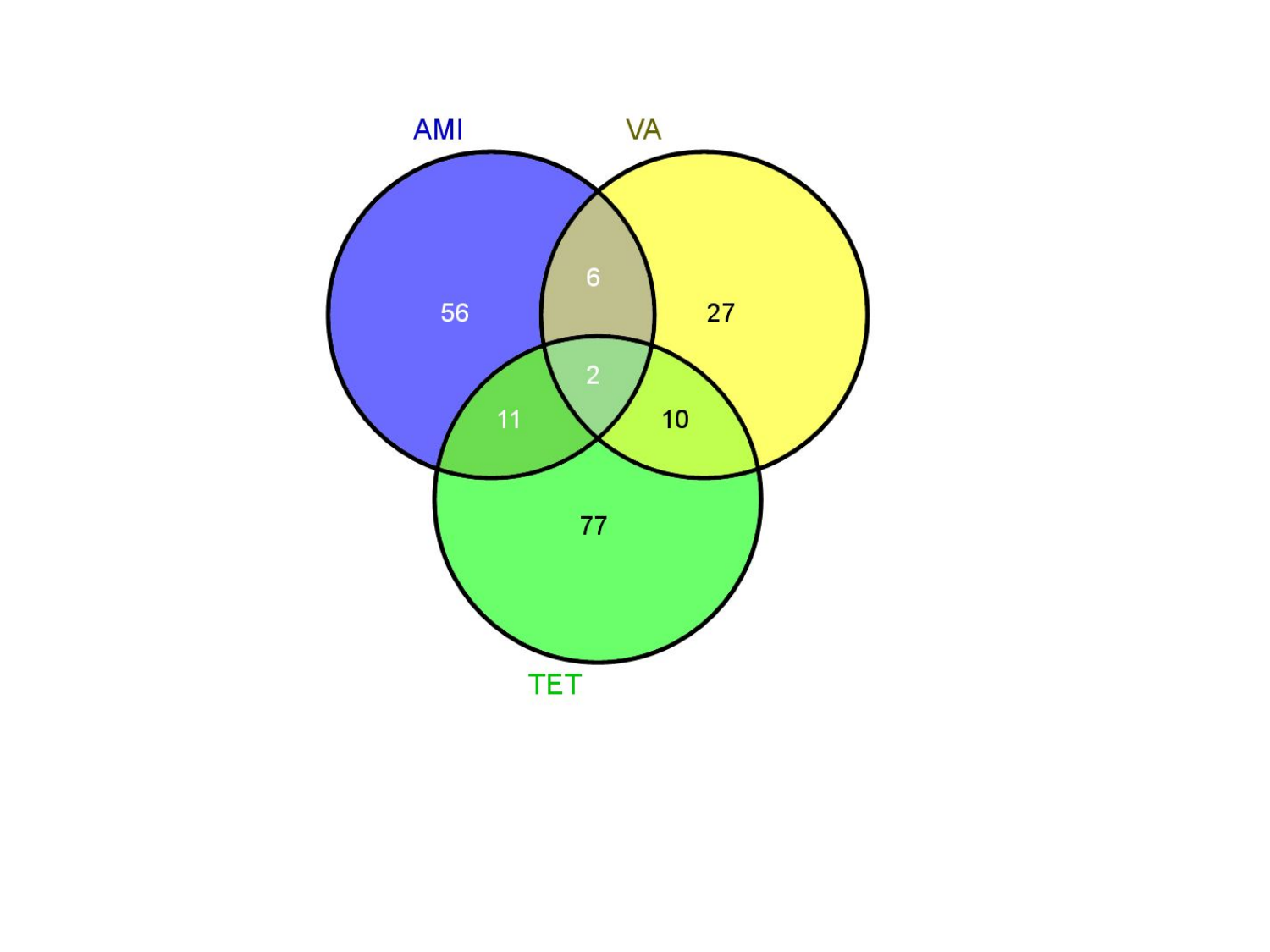

Supplement: Figure S5 — Identification of candidate biomarkers for steatogenic drugs in mouse PCLS. To identify candidate biomarkers for steatogenic drugs, significant genes found by GSEA in precision cut liver slices (PCLS) treated with amiodarone (AMI), valproic acid (VA), and tetracycline (TET) were analysed by Venn diagrams. Eight overlapping, upregulated genes in PCLS treated with AMI and VA were considered as candidate biomarkers for PPARs agonists. Genes uniquely downregulated by TET (i.e. 77), were considered as candidate biomarkers for TET-like acting compounds. (PPTX) [file pone.0086795.s005.pptx]
