# Supplementary material for: Model Steatogenic Compounds (Amiodarone, Valproic Acid, and Tetracycline) Alter Lipid Metabolism by Different Mechanisms in Mouse Liver Slices
Source: PLoS One. 2014 Jan 29;9(1):e86795. doi: 10.1371/journal.pone.0086795 (PMC3906077; doi:10.1371/journal.pone.0086795)
Supplement: Figure S4 — Effect of tetracycline on PPARα-, PPAR β/δ-, and PPARγ gene reporter assays. Luciferase activity of PPARα-, PPAR β/δ-, and PPARγ- CALUX cells on exposure to corresponding agonists GW7647 (A), L-165, 041 (D), and rosiglitazone (G) respectively. Tetracycline (TET) was tested in both agonistic (B, E, H) and antagonistic (C, F, I) modes in the 3 PPAR-CALUX assays. Data are corrected for solvent control values and are expressed as means±standard errors (n = 3). X axis represents concentration of the tested compounds [M] and y axis represents luciferase units. (PPTX) [file pone.0086795.s004.pptx]

## Slide 1
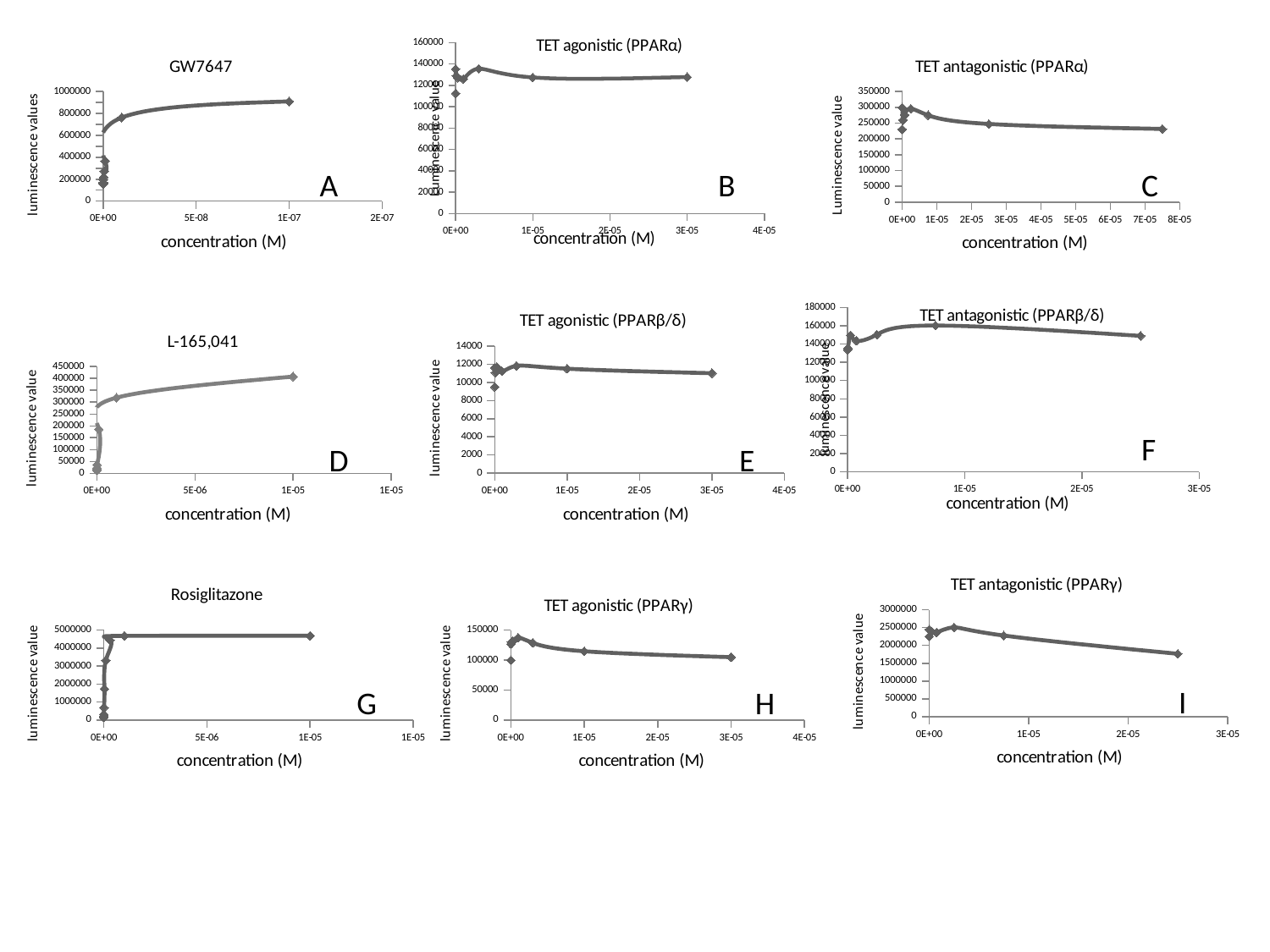

### Chart: TET agonistic (PPARα)
| Category | |
|---|---|
### Chart: GW7647
| Category | |
|---|---|
### Chart: TET antagonistic (PPARα)
| Category | |
|---|---| A
 B
 C
### Chart: TET agonistic (PPARβ/δ)
| Category | |
|---|---|
### Chart: TET antagonistic (PPARβ/δ)
| Category | |
|---|---|
### Chart: L-165,041
| Category | |
|---|---| F
 D
 E
### Chart: TET antagonistic (PPARγ)
| Category | |
|---|---|
### Chart: Rosiglitazone
| Category | |
|---|---|
### Chart: TET agonistic (PPARγ)
| Category | |
|---|---| I
 G
 H
