# Supplementary material for: Model Steatogenic Compounds (Amiodarone, Valproic Acid, and Tetracycline) Alter Lipid Metabolism by Different Mechanisms in Mouse Liver Slices
Source: PLoS One. 2014 Jan 29;9(1):e86795. doi: 10.1371/journal.pone.0086795 (PMC3906077; doi:10.1371/journal.pone.0086795)
Supplement: Table S1 — ANNI gene sets. Gene sets related to diverse hepatic and non-hepatic functions were created in ANNI and were used in GSEA to detect major biological processes affected by treatment with amiodarone, valproic acid, and tetracycline. (DOCX) [file pone.0086795.s006.docx]

**Table S1. ANNI gene sets.**

Gene sets related to diverse hepatic and non-hepatic functions were created in ANNI and were used in GSEA to detect major biological processes affected by treatment with amiodarone, valproic acid, and tetracycline. The most upper row informs about the general functional category and the lower cells contain queries used in ANNI to create presented gene sets.

| Liver functions | Energy metabolism | Toxicity and drug metabolism | Inflammation | Stress and adaptation | Others |
| --- | --- | --- | --- | --- | --- |
| Hepatocyte  Kupffer cells  Stellate cells  Fatty liver  Cholestasis  Cirrhosis  Cholesterol synthesis  Liver carcinoma  Coagulation  Fibrosis  Drug metabolism  Bile acid metabolism  ABC transporters | Lipid metabolism  FA metabolism  Mitochondrial diseases  Mitochondria  Peroxisomes  Energy metabolism  Glucose metabolism | Toxicity  Immunocytotoxicity  Drug metabolism | Regulation of NK cells functions  Regulation of lymphocyte functions  Regulation of T cells functions  Inflammation  Reactome activated TLR4 signalling | Biological adaptation to stress  Sumoylation  Extracellular matrix  Protein folding  Oxidative stress  Hypoxia  Apoptosis  Necrosis | Morphogenesis  Angiogenesis  Tight junctions  Golgi  Adipogenesis  Osteogenesis  Brain  Colon  Kidney  Heart  Reproduction |
