# Supplementary material for: Model Steatogenic Compounds (Amiodarone, Valproic Acid, and Tetracycline) Alter Lipid Metabolism by Different Mechanisms in Mouse Liver Slices
Source: PLoS One. 2014 Jan 29;9(1):e86795. doi: 10.1371/journal.pone.0086795 (PMC3906077; doi:10.1371/journal.pone.0086795)
Supplement: Table S2 — Total GO analysis. Significant genes altered by steatogenic drugs in precision cut liver slices (PCLS) were subjected to Gene Ontology (GO) analysis in DAVID. The GO analysis identified 288 (27 up- and 261-down-regulated), 152 (18 up- and 136 down-regulated), and 21 (downregulated) GO terms for amiodarone, valproic acid, and tetracycline respectively. (DOCX) [file pone.0086795.s007.docx]

**Table S2. Total GO analysis.**

Significant genes altered by steatogenic drugs in precision cut liver slices (PCLS) were subjected to Gene Ontology (GO) analysis in DAVID. The GO analysis identified 288 (27 up- and 261-down-regulated), 152 (18 up- and 136 down-regulated), and 21 (downregulated) GO terms for amiodarone, valproic acid, and tetracycline respectively.

Table S2A. Amiodarone

| Annotation Cluster 1 | Enrichment Score: 11.1 | Significant genes | Total nr of genes in GO | Benjamini | Direction of changes |
| --- | --- | --- | --- | --- | --- |
| PEROXISOME | GO:0005777~peroxisome | 15 | 78 | 1.07E-13 | up |
|  | GO:0042579~microbody | 15 | 78 | 1.07E-13 | up |
|  | GO:0031903~microbody membrane | 8 | 78 | 4.87E-09 | up |
|  | GO:0005778~peroxisomal membrane | 8 | 78 | 4.87E-09 | up |
|  | GO:0044438~microbody part | 8 | 78 | 1.27E-08 | up |
|  | GO:0044439~peroxisomal part | 8 | 78 | 1.27E-08 | up |
| Annotation Cluster 2 | Enrichment Score: 10.6 |  |  |  |  |
| MITOCHONDRION | GO:0005739~mitochondrion | 45 | 78 | 1.37E-21 | up |
|  | GO:0044429~mitochondrial part | 25 | 78 | 1.52E-13 | up |
|  | GO:0031090~organelle membrane | 24 | 78 | 7.22E-09 | up |
|  | GO:0005740~mitochondrial envelope | 17 | 78 | 2.69E-08 | up |
|  | GO:0031966~mitochondrial membrane | 15 | 78 | 7.14E-07 | up |
|  | GO:0031967~organelle envelope | 17 | 78 | 1.98E-06 | up |
|  | GO:0031975~envelope | 17 | 78 | 1.87E-06 | up |
|  | GO:0005743~mitochondrial inner membrane | 12 | 78 | 1.93E-05 | up |
|  | GO:0019866~organelle inner membrane | 12 | 78 | 2.97E-05 | up |
|  | GO:0005759~mitochondrial matrix | 10 | 78 | 7.66E-06 | up |
|  | GO:0031980~mitochondrial lumen | 10 | 78 | 7.66E-06 | up |
| Annotation Cluster 3 | Enrichment Score: 8.0 |  |  |  |  |
| FATTY ACID METABOLISM | GO:0006631~fatty acid metabolic process | 15 | 83 | 1.15E-09 | up |
|  | GO:0055114~oxidation reduction | 19 | 83 | 1.23E-05 | up |
|  | GO:0009055~electron carrier activity | 8 | 83 | 1.73E-02 | up |
| Annotation Cluster 4 | Enrichment Score: 5.5 |  |  |  |  |
| METABOLIC PROCESS | GO:0051186~cofactor metabolic process | 18 | 83 | 3.67E-13 | up |
|  | GO:0006732~coenzyme metabolic process | 16 | 83 | 2.29E-12 | up |
|  | GO:0051188~cofactor biosynthetic process | 9 | 83 | 1.45E-05 | up |
|  | GO:0009108~coenzyme biosynthetic process | 8 | 83 | 1.46E-05 | up |
| Annotation Cluster 5 | Enrichment Score: 28.3 |  |  |  |  |
| EXTRACELLULAR MATRIX | GO:0044421~extracellular region part | 106 | 504 | 2.78E-27 | down |
|  | GO:0005615~extracellular space | 80 | 504 | 8.05E-24 | down |
|  | GO:0005576~extracellular region | 156 | 504 | 2.80E-23 | down |
| Annotation Cluster 6 | Enrichment Score: 18.6 |  |  |  |  |
| ANGIOGENESIS | GO:0001944~vasculature development | 57 | 615 | 5.78E-21 | down |
|  | GO:0001568~blood vessel development | 55 | 615 | 4.78E-20 | down |
|  | GO:0048514~blood vessel morphogenesis | 43 | 615 | 6.98E-15 | down |
|  | GO:0001525~angiogenesis | 30 | 615 | 8.11E-11 | down |
| Annotation Cluster 7 | Enrichment Score: 13.5 |  |  |  |  |
| APOPTOSIS | GO:0006915~apoptosis | 48 | 670 | 4.41E-13 | down |
|  | GO:0008219~cell death | 66 | 615 | 2.27E-12 | down |
|  | GO:0012501~programmed cell death | 63 | 615 | 3.29E-12 | down |
|  | GO:0016265~death | 66 | 615 | 5.72E-12 | down |
|  | GO:0006915~apoptosis | 61 | 615 | 1.46E-11 | down |
| Annotation Cluster 8 | Enrichment Score: 13.5 |  |  |  |  |
| WOUNDING | GO:0009611~response to wounding | 60 | 615 | 2.17E-16 | down |
|  | GO:0006954~inflammatory response | 40 | 615 | 2.84E-11 | down |
|  | GO:0006952~defense response | 56 | 615 | 7.84E-10 | down |
| Annotation Cluster 9 | Enrichment Score: 10.6 |  |  |  |  |
| TRANSCRIPTION | GO:0010604~positive regulation of macromolecule metabolic process | 83 | 615 | 6.94E-16 | down |
|  | GO:0010628~positive regulation of gene expression | 69 | 615 | 1.11E-14 | down |
|  | GO:0009891~positive regulation of biosynthetic process | 74 | 615 | 2.49E-14 | down |
|  | GO:0031328~positive regulation of cellular biosynthetic process | 73 | 615 | 4.35E-14 | down |
|  | GO:0010557~positive regulation of macromolecule biosynthetic process | 71 | 615 | 6.14E-14 | down |
|  | GO:0051173~positive regulation of nitrogen compound metabolic process | 70 | 615 | 1.35E-13 | down |
|  | GO:0045935~positive regulation of nucleobase, nucleoside, nucleotide and nucleic acid metabolic process | 68 | 615 | 2.96E-13 | down |
|  | GO:0045941~positive regulation of transcription | 65 | 615 | 3.65E-13 | down |
|  | GO:0045893~positive regulation of transcription, DNA-dependent | 57 | 615 | 1.57E-11 | down |
|  | GO:0051254~positive regulation of RNA metabolic process | 57 | 615 | 2.07E-11 | down |
|  | GO:0045944~positive regulation of transcription from RNA polymerase II promoter | 51 | 615 | 6.96E-11 | down |
|  | GO:0006357~regulation of transcription from RNA polymerase II promoter | 68 | 615 | 1.01E-09 | down |
|  | GO:0003700~transcription factor activity | 75 | 576 | 1.15E-08 | down |
|  | GO:0030528~transcription regulator activity | 101 | 576 | 1.15E-08 | down |
|  | GO:0051252~regulation of RNA metabolic process | 117 | 615 | 7.39E-08 | down |
|  | GO:0006355~regulation of transcription, DNA-dependent | 112 | 615 | 7.02E-07 | down |
|  | GO:0045449~regulation of transcription | 148 | 615 | 1.27E-05 | down |
|  | GO:0043565~sequence-specific DNA binding | 50 | 576 | 7.94E-05 | down |
|  | GO:0003677~DNA binding | 117 | 576 | 1.19E-04 | down |
|  | GO:0006350~transcription | 110 | 615 | 4.78E-03 | down |
| Annotation Cluster 10 | Enrichment Score: 9.9 |  |  |  |  |
| CELL MIGRATION | GO:0016477~cell migration | 39 | 615 | 7.72E-10 | down |
|  | GO:0048870~cell motility | 41 | 615 | 7.64E-09 | down |
|  | GO:0051674~localization of cell | 41 | 615 | 7.64E-09 | down |
|  | GO:0006928~cell motion | 46 | 615 | 4.67E-08 | down |
| Annotation Cluster 11 | Enrichment Score: 9.7 |  |  |  |  |
| APOPTOSIS 2 | GO:0043067~regulation of programmed cell death | 75 | 615 | 9.83E-15 | down |
|  | GO:0010941~regulation of cell death | 75 | 615 | 1.19E-14 | down |
|  | GO:0042981~regulation of apoptosis | 74 | 615 | 1.30E-14 | down |
|  | GO:0043068~positive regulation of programmed cell death | 39 | 615 | 2.56E-09 | down |
|  | GO:0010942~positive regulation of cell death | 39 | 615 | 3.19E-09 | down |
|  | GO:0043065~positive regulation of apoptosis | 38 | 615 | 7.46E-09 | down |
|  | GO:0043069~negative regulation of programmed cell death | 34 | 615 | 5.56E-07 | down |
|  | GO:0060548~negative regulation of cell death | 34 | 615 | 6.09E-07 | down |
|  | GO:0043066~negative regulation of apoptosis | 33 | 615 | 1.09E-06 | down |
|  | GO:0012502~induction of programmed cell death | 24 | 615 | 3.84E-05 | down |
|  | GO:0006917~induction of apoptosis | 24 | 615 | 3.84E-05 | down |
| Annotation Cluster 12 | Enrichment Score: 9.6 |  |  |  |  |
| CYTOKINE ACTIVITY | GO:0005125~cytokine activity | 37 | 576 | 5.69E-12 | down |
|  | GO:0008009~chemokine activity | 17 | 576 | 4.08E-10 | down |
|  | GO:0042379~chemokine receptor binding | 17 | 576 | 4.43E-10 | down |
|  | GO:0006935~chemotaxis | 24 | 615 | 2.37E-08 | down |
|  | GO:0042330~taxis | 24 | 615 | 2.37E-08 | down |
|  | GO:0007610~behavior | 45 | 615 | 1.83E-06 | down |
| Annotation Cluster 13 | Enrichment Score: 8.3 |  |  |  |  |
| DEVELOPMENT | GO:0001501~skeletal system development | 45 | 615 | 5.47E-11 | down |
|  | GO:0001503~ossification | 23 | 615 | 6.69E-08 | down |
|  | GO:0060348~bone development | 24 | 615 | 9.36E-08 | down |
| Annotation Cluster 14 | Enrichment Score: 8.3 |  |  |  |  |
| CELL SIGNALLING | GO:0010647~positive regulation of cell communication | 37 | 615 | 1.45E-11 | down |
|  | GO:0009967~positive regulation of signal transduction | 35 | 615 | 2.08E-11 | down |
|  | GO:0010627~regulation of protein kinase cascade | 28 | 615 | 6.14E-08 | down |
|  | GO:0043408~regulation of MAPKKK cascade | 18 | 615 | 1.79E-05 | down |
|  | GO:0010740~positive regulation of protein kinase cascade | 17 | 615 | 3.78E-05 | down |
| Annotation Cluster 15 | Enrichment Score: 7.7 |  |  |  |  |
| POLYSACHCARIDE BINDING | GO:0030247~polysaccharide binding | 24 | 576 | 4.87E-07 | down |
|  | GO:0001871~pattern binding | 24 | 576 | 4.87E-07 | down |
|  | GO:0030246~carbohydrate binding | 38 | 576 | 2.98E-06 | down |
|  | GO:0008201~heparin binding | 18 | 576 | 5.31E-06 | down |
|  | GO:0005539~glycosaminoglycan binding | 21 | 576 | 5.35E-06 | down |
| Annotation Cluster 16 | Enrichment Score: 7.5 |  |  |  |  |
| DEVELOPMENT 2 | GO:0035295~tube development | 45 | 615 | 5.01E-12 | down |
|  | GO:0030323~respiratory tube development | 20 | 615 | 1.60E-05 | down |
|  | GO:0030324~lung development | 19 | 615 | 4.76E-05 | down |
|  | GO:0060541~respiratory system development | 19 | 615 | 2.08E-04 | down |
| Annotation Cluster 17 | Enrichment Score: 7.4 |  |  |  |  |
| IMMUNE SYSTEM | GO:0001775~cell activation | 42 | 615 | 2.75E-11 | down |
|  | GO:0002520~immune system development | 44 | 615 | 6.08E-10 | down |
|  | GO:0048534~hemopoietic or lymphoid organ development | 42 | 615 | 1.64E-09 | down |
|  | GO:0045321~leukocyte activation | 36 | 615 | 3.55E-09 | down |
|  | GO:0046649~lymphocyte activation | 32 | 615 | 2.51E-08 | down |
|  | GO:0030097~hemopoiesis | 35 | 615 | 3.27E-07 | down |
|  | GO:0042110~T cell activation | 21 | 615 | 5.85E-06 | down |
|  | GO:0002521~leukocyte differentiation | 23 | 615 | 9.57E-06 | down |
|  | GO:0030098~lymphocyte differentiation | 19 | 615 | 6.65E-05 | down |
|  | GO:0042113~B cell activation | 13 | 615 | 2.42E-03 | down |
| Annotation Cluster 18 | Enrichment Score: 6.5 |  |  |  |  |
| KINASE ACTIVITY | GO:0051174~regulation of phosphorus metabolic process | 46 | 615 | 8.76E-11 | down |
|  | GO:0019220~regulation of phosphate metabolic process | 46 | 615 | 8.76E-11 | down |
|  | GO:0042325~regulation of phosphorylation | 45 | 615 | 9.14E-11 | down |
|  | GO:0044093~positive regulation of molecular function | 42 | 615 | 2.02E-08 | down |
|  | GO:0045859~regulation of protein kinase activity | 31 | 615 | 5.26E-08 | down |
|  | GO:0043549~regulation of kinase activity | 31 | 615 | 9.35E-08 | down |
|  | GO:0044092~negative regulation of molecular function | 25 | 615 | 1.71E-07 | down |
|  | GO:0051338~regulation of transferase activity | 31 | 615 | 2.12E-07 | down |
|  | GO:0043086~negative regulation of catalytic activity | 20 | 615 | 3.13E-06 | down |
|  | GO:0043085~positive regulation of catalytic activity | 33 | 615 | 6.88E-06 | down |
|  | GO:0045860~positive regulation of protein kinase activity | 21 | 615 | 1.62E-05 | down |
|  | GO:0043405~regulation of MAP kinase activity | 17 | 615 | 2.09E-05 | down |
|  | GO:0033674~positive regulation of kinase activity | 21 | 615 | 3.23E-05 | down |
|  | GO:0051347~positive regulation of transferase activity | 21 | 615 | 5.51E-05 | down |
|  | GO:0033673~negative regulation of kinase activity | 12 | 615 | 2.43E-04 | down |
|  | GO:0006469~negative regulation of protein kinase activity | 12 | 615 | 2.43E-04 | down |
|  | GO:0051348~negative regulation of transferase activity | 12 | 615 | 3.37E-04 | down |
|  | GO:0000165~MAPKKK cascade | 16 | 615 | 2.43E-03 | down |
|  | GO:0043406~positive regulation of MAP kinase activity | 11 | 615 | 3.54E-03 | down |
| Annotation Cluster 19 | Enrichment Score: 6.1 |  |  |  |  |
| IMMUNE CELLS ACTIVITY | GO:0050865~regulation of cell activation | 35 | 615 | 1.55E-12 | down |
|  | GO:0002694~regulation of leukocyte activation | 34 | 615 | 5.48E-12 | down |
|  | GO:0051249~regulation of lymphocyte activation | 31 | 615 | 1.07E-10 | down |
|  | GO:0002684~positive regulation of immune system process | 36 | 615 | 6.91E-10 | down |
|  | GO:0050867~positive regulation of cell activation | 24 | 615 | 5.35E-09 | down |
|  | GO:0051251~positive regulation of lymphocyte activation | 23 | 615 | 7.49E-09 | down |
|  | GO:0002696~positive regulation of leukocyte activation | 23 | 615 | 2.01E-08 | down |
|  | GO:0050670~regulation of lymphocyte proliferation | 20 | 615 | 7.53E-08 | down |
|  | GO:0032944~regulation of mononuclear cell proliferation | 20 | 615 | 7.53E-08 | down |
|  | GO:0050863~regulation of T cell activation | 23 | 615 | 8.85E-08 | down |
|  | GO:0070663~regulation of leukocyte proliferation | 20 | 615 | 1.08E-07 | down |
|  | GO:0030888~regulation of B cell proliferation | 12 | 615 | 2.37E-06 | down |
|  | GO:0002683~negative regulation of immune system process | 17 | 615 | 5.72E-06 | down |
|  | GO:0050864~regulation of B cell activation | 14 | 615 | 1.72E-05 | down |
|  | GO:0050870~positive regulation of T cell activation | 15 | 615 | 2.51E-05 | down |
|  | GO:0050671~positive regulation of lymphocyte proliferation | 13 | 615 | 3.58E-05 | down |
|  | GO:0032946~positive regulation of mononuclear cell proliferation | 13 | 615 | 3.58E-05 | down |
|  | GO:0070665~positive regulation of leukocyte proliferation | 13 | 615 | 5.30E-05 | down |
|  | GO:0002695~negative regulation of leukocyte activation | 13 | 615 | 6.40E-05 | down |
|  | GO:0050866~negative regulation of cell activation | 13 | 615 | 6.40E-05 | down |
|  | GO:0051250~negative regulation of lymphocyte activation | 12 | 615 | 2.89E-04 | down |
|  | GO:0050871~positive regulation of B cell activation | 10 | 615 | 4.73E-04 | down |
|  | GO:0030890~positive regulation of B cell proliferation | 8 | 615 | 5.46E-04 | down |
|  | GO:0050672~negative regulation of lymphocyte proliferation | 9 | 615 | 1.82E-03 | down |
|  | GO:0032945~negative regulation of mononuclear cell proliferation | 9 | 615 | 1.82E-03 | down |
|  | GO:0070664~negative regulation of leukocyte proliferation | 9 | 615 | 1.82E-03 | down |
|  | GO:0045619~regulation of lymphocyte differentiation | 11 | 615 | 3.11E-03 | down |
|  | GO:0042129~regulation of T cell proliferation | 11 | 615 | 3.54E-03 | down |
|  | GO:0050869~negative regulation of B cell activation | 6 | 615 | 8.25E-03 | down |
| Annotation Cluster 20 | Enrichment Score: 6.0 |  |  |  |  |
| ANTIMICROBIAL ACTIVITY | GO:0002237~response to molecule of bacterial origin | 15 | 615 | 6.71E-07 | down |
|  | GO:0032496~response to lipopolysaccharide | 11 | 615 | 9.58E-05 | down |
|  | GO:0009617~response to bacterium | 22 | 615 | 1.50E-04 | down |
| Annotation Cluster 21 | Enrichment Score: 5.9 |  |  |  |  |
| CELL ADHESION | GO:0007155~cell adhesion | 55 | 615 | 3.23E-06 | down |
|  | GO:0022610~biological adhesion | 55 | 615 | 3.40E-06 | down |
|  | GO:0016337~cell-cell adhesion | 25 | 615 | 2.33E-03 | down |
| Annotation Cluster 22 | Enrichment Score: 5.8 |  |  |  |  |
| DEVELOPMENT 3 | GO:0060429~epithelium development | 38 | 615 | 6.99E-08 | down |
|  | GO:0048729~tissue morphogenesis | 33 | 615 | 9.93E-07 | down |
|  | GO:0001763~morphogenesis of a branching structure | 23 | 615 | 1.19E-06 | down |
|  | GO:0035239~tube morphogenesis | 25 | 615 | 1.77E-05 | down |
|  | GO:0002009~morphogenesis of an epithelium | 24 | 615 | 6.60E-05 | down |
| Annotation Cluster 23 | Enrichment Score: 5.5 |  |  |  |  |
| TRANSCRIPTION 2 | GO:0051098~regulation of binding | 21 | 615 | 9.39E-08 | down |
|  | GO:0051101~regulation of DNA binding | 17 | 615 | 3.96E-06 | down |
|  | GO:0051090~regulation of transcription factor activity | 15 | 615 | 6.63E-06 | down |
|  | GO:0051099~positive regulation of binding | 12 | 615 | 7.21E-05 | down |
|  | GO:0051092~positive regulation of NF-kappaB transcription factor activity | 9 | 615 | 1.70E-04 | down |
|  | GO:0043388~positive regulation of DNA binding | 11 | 615 | 2.40E-04 | down |
|  | GO:0051091~positive regulation of transcription factor activity | 10 | 615 | 2.53E-04 | down |
| Annotation Cluster 24 | Enrichment Score: 5.4 |  |  |  |  |
| DEVELOPMENT 4 | GO:0048598~embryonic morphogenesis | 41 | 615 | 3.48E-06 | down |
|  | GO:0007389~pattern specification process | 35 | 615 | 5.45E-06 | down |
|  | GO:0009952~anterior/posterior pattern formation | 21 | 615 | 3.14E-04 | down |
|  | GO:0003002~regionalization | 23 | 615 | 3.53E-03 | down |
| Annotation Cluster 25 | Enrichment Score: 5.2 |  |  |  |  |
| CELL MOTION | GO:0051270~regulation of cell motion | 23 | 615 | 7.45E-08 | down |
|  | GO:0030334~regulation of cell migration | 19 | 615 | 3.46E-06 | down |
|  | GO:0040012~regulation of locomotion | 20 | 615 | 1.06E-05 | down |
|  | GO:0051272~positive regulation of cell motion | 10 | 615 | 1.05E-03 | down |
| Annotation Cluster 26 | Enrichment Score: 4.9 |  |  |  |  |
| DEVELOPMENT 5 | GO:0043009~chordate embryonic development | 50 | 615 | 4.79E-08 | down |
|  | GO:0009792~embryonic development ending in birth or egg hatching | 50 | 615 | 6.37E-08 | down |
|  | GO:0001701~in utero embryonic development | 35 | 615 | 1.41E-06 | down |
|  | GO:0048598~embryonic morphogenesis | 41 | 615 | 3.48E-06 | down |
|  | GO:0048568~embryonic organ development | 32 | 615 | 3.87E-06 | down |
|  | GO:0001892~embryonic placenta development | 13 | 615 | 2.64E-04 | down |
|  | GO:0001890~placenta development | 14 | 615 | 1.85E-03 | down |
|  | GO:0060711~labyrinthine layer development | 8 | 615 | 9.22E-03 | down |
| Annotation Cluster 27 | Enrichment Score: 4.7 |  |  |  |  |
| HORMONAL RESPONSE | GO:0010033~response to organic substance | 65 | 615 | 5.40E-12 | down |
|  | GO:0009719~response to endogenous stimulus | 32 | 615 | 1.06E-08 | down |
|  | GO:0009725~response to hormone stimulus | 29 | 615 | 5.72E-08 | down |
| Annotation Cluster 28 | Enrichment Score: 4.1 |  |  |  |  |
| KINASE ACTIVITY 2 | GO:0006468~protein amino acid phosphorylation | 65 | 615 | 6.70E-08 | down |
|  | GO:0006793~phosphorus metabolic process | 78 | 615 | 2.35E-07 | down |
|  | GO:0006796~phosphate metabolic process | 78 | 615 | 2.35E-07 | down |
|  | GO:0016310~phosphorylation | 66 | 615 | 1.71E-06 | down |
|  | GO:0004672~protein kinase activity | 52 | 576 | 6.38E-05 | down |
|  | GO:0004713~protein tyrosine kinase activity | 21 | 576 | 9.87E-04 | down |
|  | GO:0004714~transmembrane receptor protein tyrosine kinase activity | 11 | 576 | 3.93E-03 | down |
| Annotation Cluster 29 | Enrichment Score: 4.1 |  |  |  |  |
| IMMUNE CELLS ACTIVITY 2 | GO:0070661~leukocyte proliferation | 11 | 615 | 2.88E-04 | down |
|  | GO:0032943~mononuclear cell proliferation | 11 | 615 | 2.88E-04 | down |
|  | GO:0046651~lymphocyte proliferation | 10 | 615 | 1.26E-03 | down |
| Annotation Cluster 30 | Enrichment Score: 4.0 |  |  |  |  |
| TRANSCRIPTION 3 | GO:0010605~negative regulation of macromolecule metabolic process | 49 | 615 | 2.08E-05 | down |
|  | GO:0031327~negative regulation of cellular biosynthetic process | 40 | 615 | 4.40E-04 | down |
|  | GO:0010558~negative regulation of macromolecule biosynthetic process | 39 | 615 | 5.14E-04 | down |
|  | GO:0009890~negative regulation of biosynthetic process | 40 | 615 | 5.23E-04 | down |
|  | GO:0051172~negative regulation of nitrogen compound metabolic process | 37 | 615 | 1.02E-03 | down |
|  | GO:0016481~negative regulation of transcription | 35 | 615 | 1.12E-03 | down |
|  | GO:0010629~negative regulation of gene expression | 37 | 615 | 1.51E-03 | down |
|  | GO:0045934~negative regulation of nucleobase, nucleoside, nucleotide and nucleic acid metabolic process | 36 | 615 | 1.72E-03 | down |
|  | GO:0045892~negative regulation of transcription, DNA-dependent | 30 | 615 | 2.07E-03 | down |
|  | GO:0051253~negative regulation of RNA metabolic process | 30 | 615 | 2.28E-03 | down |
| Annotation Cluster 31 | Enrichment Score: 4.0 |  |  |  |  |
| IMMUNE RESPONSE | GO:0002684~positive regulation of immune system process | 36 | 615 | 6.91E-10 | down |
|  | GO:0048584~positive regulation of response to stimulus | 27 | 615 | 7.13E-06 | down |
|  | GO:0050778~positive regulation of immune response | 21 | 615 | 6.11E-05 | down |
|  | GO:0002253~activation of immune response | 15 | 615 | 4.61E-04 | down |
|  | GO:0002757~immune response-activating signal transduction | 11 | 615 | 7.03E-04 | down |
|  | GO:0002764~immune response-regulating signal transduction | 11 | 615 | 1.35E-03 | down |
| Annotation Cluster 32 | Enrichment Score: 3.8 |  |  |  |  |
| NUCLEOPLASM | GO:0005654~nucleoplasm | 50 | 504 | 7.06E-05 | down |
|  | GO:0044451~nucleoplasm part | 45 | 504 | 6.28E-05 | down |
|  | GO:0005667~transcription factor complex | 27 | 504 | 8.99E-05 | down |
|  | GO:0031981~nuclear lumen | 57 | 504 | 9.75E-03 | down |
| Annotation Cluster 33 | Enrichment Score: 3.6 |  |  |  |  |
| KINASE ACTIVITY 3 | GO:0032268~regulation of cellular protein metabolic process | 31 | 615 | 1.92E-04 | down |
|  | GO:0031399~regulation of protein modification process | 22 | 615 | 3.00E-04 | down |
|  | GO:0001932~regulation of protein amino acid phosphorylation | 18 | 615 | 4.77E-04 | down |
|  | GO:0010562~positive regulation of phosphorus metabolic process | 12 | 615 | 1.32E-03 | down |
|  | GO:0045937~positive regulation of phosphate metabolic process | 12 | 615 | 1.32E-03 | down |
|  | GO:0051247~positive regulation of protein metabolic process | 16 | 615 | 1.56E-03 | down |
|  | GO:0032270~positive regulation of cellular protein metabolic process | 15 | 615 | 2.10E-03 | down |
|  | GO:0031401~positive regulation of protein modification process | 13 | 615 | 2.42E-03 | down |
|  | GO:0042327~positive regulation of phosphorylation | 11 | 615 | 4.00E-03 | down |
| Annotation Cluster 34 | Enrichment Score: 3.4 |  |  |  |  |
| PROTEIN TRANSPORT | GO:0051050~positive regulation of transport | 24 | 615 | 7.34E-07 | down |
|  | GO:0060341~regulation of cellular localization | 25 | 615 | 6.26E-06 | down |
|  | GO:0051223~regulation of protein transport | 15 | 615 | 2.11E-05 | down |
|  | GO:0032880~regulation of protein localization | 17 | 615 | 4.98E-05 | down |
|  | GO:0070201~regulation of establishment of protein localization | 15 | 615 | 5.79E-05 | down |
|  | GO:0051222~positive regulation of protein transport | 10 | 615 | 3.86E-04 | down |
|  | GO:0051047~positive regulation of secretion | 11 | 615 | 1.56E-03 | down |
| Annotation Cluster 35 | Enrichment Score: 3.3 |  |  |  |  |
| REPRODUCTION | GO:0003006~reproductive developmental process | 34 | 615 | 3.18E-06 | down |
|  | GO:0032504~multicellular organism reproduction | 41 | 615 | 7.04E-05 | down |
|  | GO:0048609~reproductive process in a multicellular organism | 41 | 615 | 7.04E-05 | down |
| Annotation Cluster 36 | Enrichment Score: 2.9 |  |  |  |  |
| MESENCHYMAL CELLS | GO:0048762~mesenchymal cell differentiation | 11 | 615 | 8.35E-04 | down |
|  | GO:0060485~mesenchyme development | 11 | 615 | 9.88E-04 | down |
|  | GO:0014031~mesenchymal cell development | 10 | 615 | 2.80E-03 | down |
| Annotation Cluster 37 | Enrichment Score: 2.8 |  |  |  |  |
| IMMUNE CELLS ACTIVITY 3 | GO:0050863~regulation of T cell activation | 23 | 615 | 8.85E-08 | down |
|  | GO:0050870~positive regulation of T cell activation | 15 | 615 | 2.51E-05 | down |
|  | GO:0050671~positive regulation of lymphocyte proliferation | 13 | 615 | 3.58E-05 | down |
|  | GO:0032946~positive regulation of mononuclear cell proliferation | 13 | 615 | 3.58E-05 | down |
|  | GO:0070665~positive regulation of leukocyte proliferation | 13 | 615 | 5.30E-05 | down |
|  | GO:0045619~regulation of lymphocyte differentiation | 11 | 615 | 3.11E-03 | down |
| Annotation Cluster 38 | Enrichment Score: 2.8 |  |  |  |  |
| CALCIUM HOMEOSTASIS | GO:0042592~homeostatic process | 54 | 615 | 2.14E-05 | down |
|  | GO:0006875~cellular metal ion homeostasis | 15 | 615 | 2.10E-03 | down |
|  | GO:0006874~cellular calcium ion homeostasis | 14 | 615 | 2.80E-03 | down |
|  | GO:0055065~metal ion homeostasis | 15 | 615 | 3.65E-03 | down |
|  | GO:0055074~calcium ion homeostasis | 14 | 615 | 4.11E-03 | down |
| Annotation Cluster 39 | Enrichment Score: 2.2 |  |  |  |  |
| DEVELOPMENT 6 | GO:0010721~negative regulation of cell development | 12 | 615 | 2.31E-05 | down |
|  | GO:0050768~negative regulation of neurogenesis | 11 | 615 | 9.58E-05 | down |
|  | GO:0060284~regulation of cell development | 19 | 615 | 3.81E-03 | down |

Table S2B. Valproic acid

| Annotation Cluster 1 | Enrichment Score: 4.4 | Significant genes | Total nr of genes in GO | Benjamini | Direction of changes |
| --- | --- | --- | --- | --- | --- |
| PEROXISOME | GO:0005777~peroxisome | 8 | 37 | 9.69E-07 | up |
|  | GO:0042579~microbody | 8 | 37 | 9.69E-07 | up |
|  | GO:0006631~fatty acid metabolic process | 8 | 37 | 1.67E-04 | up |
| Annotation Cluster 2 | Enrichment Score: 4.1 |  |  |  |  |
| LIPID HOMEOSTASIS | GO:0055088~lipid homeostasis | 5 | 37 | 3.41E-04 | up |
|  | GO:0006641~triglyceride metabolic process | 5 | 37 | 2.88E-04 | up |
|  | GO:0006639~acylglycerol metabolic process | 5 | 37 | 4.46E-04 | up |
|  | GO:0006638~neutral lipid metabolic process | 5 | 37 | 4.29E-04 | up |
|  | GO:0006662~glycerol ether metabolic process | 5 | 37 | 4.29E-04 | up |
|  | GO:0008202~steroid metabolic process | 6 | 37 | 2.30E-03 | up |
|  | GO:0044242~cellular lipid catabolic process | 5 | 37 | 7.40E-04 | up |
|  | GO:0016042~lipid catabolic process | 6 | 37 | 1.20E-03 | up |
|  | GO:0008202~steroid metabolic process | 6 | 37 | 2.30E-03 | up |
| Annotation Cluster 3 | Enrichment Score: 2.9 |  |  |  |  |
| MICROSOME | GO:0005792~microsome | 8 | 37 | 1.81E-05 | up |
|  | GO:0042598~vesicular fraction | 8 | 37 | 1.51E-05 | up |
|  | GO:0000267~cell fraction | 10 | 37 | 5.57E-04 | up |
|  | GO:0005624~membrane fraction | 9 | 37 | 8.38E-04 | up |
|  | GO:0005626~insoluble fraction | 9 | 37 | 8.01E-04 | up |
| Annotation Cluster 4 | Enrichment Score: 13.9 |  |  |  |  |
| TRANSCRIPTION | GO:0045449~regulation of transcription | 96 | 279 | 5.96E-11 | down |
|  | GO:0051252~regulation of RNA metabolic process | 72 | 279 | 5.16E-10 | down |
|  | GO:0006355~regulation of transcription, DNA-dependent | 71 | 279 | 6.45E-10 | down |
|  | GO:0006350~transcription | 78 | 279 | 3.00E-09 | down |
|  | GO:0030528~transcription regulator activity | 74 | 257 | 4.87E-17 | down |
|  | GO:0003700~transcription factor activity | 56 | 257 | 2.32E-15 | down |
|  | GO:0003677~DNA binding | 80 | 257 | 1.72E-11 | down |
|  | GO:0043565~sequence-specific DNA binding | 40 | 257 | 1.34E-10 | down |
| Annotation Cluster 5 | Enrichment Score: 13.2 |  |  |  |  |
| APOPTOSIS | GO:0012501~programmed cell death | 41 | 279 | 1.17E-11 | down |
|  | GO:0008219~cell death | 42 | 279 | 1.83E-11 | down |
|  | GO:0006915~apoptosis | 40 | 279 | 2.05E-11 | down |
|  | GO:0016265~death | 42 | 279 | 2.69E-11 | down |
| Annotation Cluster 6 | Enrichment Score: 11.5 |  |  |  |  |
| BIOLOGICAL PROCESS | GO:0030528~transcription regulator activity | 74 | 257 | 4.87E-17 | down |
|  | GO:0003700~transcription factor activity | 56 | 257 | 2.32E-15 | down |
|  | GO:0031328~positive regulation of cellular biosynthetic process | 42 | 279 | 1.24E-10 | down |
|  | GO:0009891~positive regulation of biosynthetic process | 42 | 279 | 1.39E-10 | down |
|  | GO:0051173~positive regulation of nitrogen compound metabolic process | 40 | 279 | 3.95E-10 | down |
|  | GO:0010604~positive regulation of macromolecule metabolic process | 44 | 279 | 4.30E-10 | down |
|  | GO:0051252~regulation of RNA metabolic process | 72 | 279 | 5.16E-10 | down |
|  | GO:0006355~regulation of transcription, DNA-dependent | 71 | 279 | 6.45E-10 | down |
|  | GO:0045941~positive regulation of transcription | 37 | 279 | 1.04E-09 | down |
|  | GO:0010557~positive regulation of macromolecule biosynthetic process | 39 | 279 | 1.32E-09 | down |
|  | GO:0045935~positive regulation of nucleobase, nucleoside, nucleotide and nucleic acid metabolic process | 38 | 279 | 1.72E-09 | down |
|  | GO:0006357~regulation of transcription from RNA polymerase II promoter | 42 | 279 | 1.75E-09 | down |
|  | GO:0010628~positive regulation of gene expression | 37 | 279 | 1.80E-09 | down |
|  | GO:0045893~positive regulation of transcription, DNA-dependent | 34 | 279 | 1.86E-09 | down |
|  | GO:0051254~positive regulation of RNA metabolic process | 34 | 279 | 2.10E-09 | down |
|  | GO:0045944~positive regulation of transcription from RNA polymerase II promoter | 30 | 279 | 1.75E-08 | down |
|  | GO:0016563~transcription activator activity | 19 | 257 | 1.14E-04 | down |
| Annotation Cluster 7 | Enrichment Score: 9.9 |  |  |  |  |
| DEFENSE RESPONSE | GO:0006952~defense response | 38 | 279 | 8.72E-11 | down |
|  | GO:0006954~inflammatory response | 27 | 279 | 1.50E-10 | down |
|  | GO:0009611~response to wounding | 31 | 279 | 1.86E-09 | down |
|  | GO:0006935~chemotaxis | 16 | 279 | 3.41E-07 | down |
|  | GO:0042330~taxis | 16 | 279 | 3.41E-07 | down |
|  | GO:0007610~behavior | 23 | 279 | 7.49E-04 | down |
|  | GO:0007626~locomotory behavior | 17 | 279 | 7.86E-04 | down |
|  | GO:0005615~extracellular space | 36 | 194 | 1.54E-11 | down |
|  | GO:0008009~chemokine activity | 16 | 257 | 1.53E-14 | down |
|  | GO:0042379~chemokine receptor binding | 16 | 257 | 2.30E-14 | down |
|  | GO:0005125~cytokine activity | 22 | 257 | 2.18E-09 | down |
| Annotation Cluster 8 | Enrichment Score: 8.0 |  |  |  |  |
| EXTRACELLULAR MATRIX | GO:0005615~extracellular space | 36 | 194 | 1.54E-11 | down |
|  | GO:0044421~extracellular region part | 41 | 194 | 9.25E-10 | down |
|  | GO:0005576~extracellular region | 56 | 194 | 1.73E-06 | down |
|  | GO:0005125~cytokine activity | 22 | 257 | 2.18E-09 | down |
| Annotation Cluster 9 | Enrichment Score: 7.1 |  |  |  |  |
| METABOLIC PROCESS | GO:0006357~regulation of transcription from RNA polymerase II promoter | 42 | 279 | 1.75E-09 | down |
|  | GO:0010605~negative regulation of macromolecule metabolic process | 34 | 279 | 2.35E-07 | down |
|  | GO:0009890~negative regulation of biosynthetic process | 31 | 279 | 3.27E-07 | down |
|  | GO:0045934~negative regulation of nucleobase, nucleoside, nucleotide and nucleic acid metabolic process | 29 | 279 | 6.42E-07 | down |
|  | GO:0051172~negative regulation of nitrogen compound metabolic process | 29 | 279 | 7.85E-07 | down |
|  | GO:0031327~negative regulation of cellular biosynthetic process | 30 | 279 | 8.98E-07 | down |
|  | GO:0010558~negative regulation of macromolecule biosynthetic process | 29 | 279 | 1.78E-06 | down |
|  | GO:0045892~negative regulation of transcription, DNA-dependent | 24 | 279 | 3.86E-06 | down |
|  | GO:0051253~negative regulation of RNA metabolic process | 24 | 279 | 4.03E-06 | down |
|  | GO:0016481~negative regulation of transcription | 26 | 279 | 6.81E-06 | down |
|  | GO:0010629~negative regulation of gene expression | 26 | 279 | 3.39E-05 | down |
|  | GO:0000122~negative regulation of transcription from RNA polymerase II promoter | 18 | 279 | 1.49E-04 | down |
| Annotation Cluster 10 | Enrichment Score: 6.6 |  |  |  |  |
| APOPTOSIS 2 | GO:0042981~regulation of apoptosis | 41 | 279 | 4.07E-10 | down |
|  | GO:0043067~regulation of programmed cell death | 41 | 279 | 5.30E-10 | down |
|  | GO:0010941~regulation of cell death | 41 | 279 | 5.59E-10 | down |
|  | GO:0043065~positive regulation of apoptosis | 22 | 279 | 1.93E-06 | down |
|  | GO:0043068~positive regulation of programmed cell death | 22 | 279 | 2.11E-06 | down |
|  | GO:0010942~positive regulation of cell death | 22 | 279 | 2.32E-06 | down |
| Annotation Cluster 11 | Enrichment Score: 6.3 |  |  |  |  |
| DEVELOPMENT | GO:0060429~epithelium development | 30 | 279 | 6.72E-11 | down |
|  | GO:0048729~tissue morphogenesis | 21 | 279 | 3.86E-06 | down |
|  | GO:0035295~tube development | 22 | 279 | 4.48E-06 | down |
|  | GO:0002009~morphogenesis of an epithelium | 17 | 279 | 1.68E-05 | down |
| Annotation Cluster 12 | Enrichment Score: 5.9 |  |  |  |  |
| DEVELOPMENT 2 | GO:0007389~pattern specification process | 26 | 279 | 5.61E-08 | down |
|  | GO:0043009~chordate embryonic development | 29 | 279 | 1.97E-06 | down |
|  | GO:0009792~embryonic development ending in birth or egg hatching | 29 | 279 | 2.30E-06 | down |
|  | GO:0048598~embryonic morphogenesis | 26 | 279 | 3.90E-06 | down |
|  | GO:0003002~regionalization | 19 | 279 | 1.37E-05 | down |
|  | GO:0009952~anterior/posterior pattern formation | 16 | 279 | 1.79E-05 | down |
| Annotation Cluster 13 | Enrichment Score: 4.5 |  |  |  |  |
| MORPHOGENESIS | GO:0048598~embryonic morphogenesis | 26 | 279 | 3.90E-06 | down |
|  | GO:0048568~embryonic organ development | 21 | 279 | 4.45E-06 | down |
|  | GO:0048562~embryonic organ morphogenesis | 16 | 279 | 3.25E-05 | down |
|  | GO:0048705~skeletal system morphogenesis | 14 | 279 | 7.24E-05 | down |
| Annotation Cluster 14 | Enrichment Score: 3.7 |  |  |  |  |
| IMMUNE SYSTEM | GO:0002520~immune system development | 23 | 279 | 6.52E-06 | down |
|  | GO:0048534~hemopoietic or lymphoid organ development | 22 | 279 | 1.13E-05 | down |
|  | GO:0030097~hemopoiesis | 20 | 279 | 3.04E-05 | down |
|  | GO:0002521~leukocyte differentiation | 13 | 279 | 8.13E-04 | down |
|  | GO:0045321~leukocyte activation | 16 | 279 | 1.01E-03 | down |
|  | GO:0001775~cell activation | 17 | 279 | 1.05E-03 | down |
|  | GO:0030099~myeloid cell differentiation | 10 | 279 | 2.94E-03 | down |
|  | GO:0046649~lymphocyte activation | 14 | 279 | 2.92E-03 | down |
| Annotation Cluster 15 | Enrichment Score: 3.0 |  |  |  |  |
| KINASE ACTIVITY | GO:0044093~positive regulation of molecular function | 23 | 279 | 1.14E-05 | down |
|  | GO:0007243~protein kinase cascade | 20 | 279 | 1.30E-05 | down |
|  | GO:0043085~positive regulation of catalytic activity | 18 | 279 | 6.66E-04 | down |
|  | GO:0042325~regulation of phosphorylation | 17 | 279 | 5.41E-03 | down |
| Annotation Cluster 16 | Enrichment Score: 2.9 |  |  |  |  |
| BIOLOGICAL RESPONSE | GO:0051098~regulation of binding | 14 | 279 | 1.74E-06 | down |
|  | GO:0051090~regulation of transcription factor activity | 11 | 279 | 1.00E-05 | down |
|  | GO:0051101~regulation of DNA binding | 11 | 279 | 7.06E-05 | down |
|  | GO:0048584~positive regulation of response to stimulus | 15 | 279 | 7.05E-04 | down |
|  | GO:0002237~response to molecule of bacterial origin | 8 | 279 | 1.23E-03 | down |
|  | GO:0044092~negative regulation of molecular function | 12 | 279 | 1.70E-03 | down |
|  | GO:0002684~positive regulation of immune system process | 15 | 279 | 1.77E-03 | down |
|  | GO:0051092~positive regulation of NF-kappaB transcription factor activity | 6 | 279 | 2.60E-03 | down |
|  | GO:0051100~negative regulation of binding | 6 | 279 | 5.47E-03 | down |
| Annotation Cluster 17 | Enrichment Score: 2.8 |  |  |  |  |
| BONE DEVELOPMENT | GO:0001503~ossification | 11 | 279 | 1.31E-03 | down |
|  | GO:0009880~embryonic pattern specification | 7 | 279 | 1.44E-03 | down |
|  | GO:0060348~bone development | 11 | 279 | 2.91E-03 | down |
|  | GO:0045667~regulation of osteoblast differentiation | 6 | 279 | 2.99E-03 | down |
| Annotation Cluster 18 | Enrichment Score: 2.8 |  |  |  |  |
| DEVELOPMENT | GO:0048596~embryonic camera-type eye morphogenesis | 5 | 279 | 7.82E-04 | down |
|  | GO:0048048~embryonic eye morphogenesis | 5 | 279 | 2.86E-03 | down |
|  | GO:0031076~embryonic camera-type eye development | 5 | 279 | 3.54E-03 | down |
|  | GO:0001654~eye development | 10 | 279 | 4.96E-02 | down |
| Annotation Cluster 19 | Enrichment Score: 2.6 |  |  |  |  |
| DEVELOPMENT | GO:0048732~gland development | 15 | 279 | 1.18E-03 | down |
|  | GO:0048608~reproductive structure development | 12 | 279 | 1.51E-03 | down |
|  | GO:0001655~urogenital system development | 12 | 279 | 3.65E-03 | down |
| Annotation Cluster 20 | Enrichment Score: 2.5 |  |  |  |  |
| ANGIOGENESIS | GO:0007507~heart development | 18 | 279 | 9.39E-05 | down |
|  | GO:0001568~blood vessel development | 16 | 279 | 2.93E-03 | down |
|  | GO:0001944~vasculature development | 16 | 279 | 3.54E-03 | down |

Table S2C. Tetracycline

| Annotation cluster 1 | Enrichment Score: 5.2 | Significant genes | Total nr of genes in GO | Benjamini | Direction of changes |
| --- | --- | --- | --- | --- | --- |
| MITOCHONDRION | GO:0005739~mitochondrion | 46 | 111 | 6.97E-09 | down |
|  | GO:0044429~mitochondrial part | 27 | 111 | 2.04E-07 | down |
|  | GO:0005759~mitochondrial matrix | 12 | 111 | 1.43E-03 | down |
|  | GO:0031980~mitochondrial lumen | 12 | 111 | 1.43E-03 | down |
| Annotation cluster 2 | Enrichment Score: 4.6 |  |  |  |  |
| MITOCHONDRION 2 | GO:0044429~mitochondrial part | 27 | 111 | 2.04E-07 | down |
|  | GO:0005743~mitochondrial inner membrane | 15 | 111 | 6.76E-04 | down |
|  | GO:0019866~organelle inner membrane | 15 | 111 | 8.16E-04 | down |
|  | GO:0005740~mitochondrial envelope | 16 | 111 | 1.56E-03 | down |
|  | GO:0031966~mitochondrial membrane | 15 | 111 | 2.99E-03 | down |
| Annotation cluster 3 | Enrichment Score: 4.6 |  |  |  |  |
| METABOLIC PROCESS | GO:0051186~cofactor metabolic process | 14 | 115 | 3.36E-04 | down |
|  | GO:0006732~coenzyme metabolic process | 12 | 115 | 4.72E-04 | down |
| Annotation cluster 4 | Enrichment Score: 4.2 |  |  |  |  |
| COFACTOR BINDING | GO:0048037~cofactor binding | 20 | 112 | 2.26E-09 | down |
|  | GO:0019842~vitamin binding | 10 | 112 | 1.88E-03 | down |
|  | GO:0070279~vitamin B6 binding | 8 | 112 | 1.68E-03 | down |
|  | GO:0030170~pyridoxal phosphate binding | 8 | 112 | 1.68E-03 | down |
| Annotation cluster 5 | Enrichment Score: 3.1 |  |  |  |  |
| ELECTRON CARRIER ACTIVITY | GO:0009055~electron carrier activity | 12 | 112 | 5.09E-05 | down |
|  | GO:0046906~tetrapyrrole binding | 9 | 112 | 1.19E-03 | down |
|  | GO:0020037~heme binding | 8 | 112 | 3.94E-03 | down |
|  | GO:0005506~iron ion binding | 13 | 112 | 4.17E-03 | down |
| Annotation cluster 6 | Enrichment Score: 2.6 |  |  |  |  |
| VITAMINE B6 BINDING | GO:0070279~vitamin B6 binding | 8 | 112 | 1.68E-03 | down |
