# Supplementary material for: Model Steatogenic Compounds (Amiodarone, Valproic Acid, and Tetracycline) Alter Lipid Metabolism by Different Mechanisms in Mouse Liver Slices
Source: PLoS One. 2014 Jan 29;9(1):e86795. doi: 10.1371/journal.pone.0086795 (PMC3906077; doi:10.1371/journal.pone.0086795)
Supplement: Table S3 — Candidate biomarkers. Functions of candidate biomarkers for PPAR agonists and tetracycline (TET)-like acting compounds are derived from GeneCards http://www.genecards.org. (DOCX) [file pone.0086795.s008.docx]

**Table S3.** Functions of candidate biomarkers for PPAR agonists and tetracycline (TET)-like acting compounds are derived from GeneCards (http://www.genecards.org).

| Gene | Function | Candidate Biomarkers |
| --- | --- | --- |
| *Abcd3* | The protein encoded by this gene is a member of the superfamily of ATP-binding cassette (ABC) transporters. ABC genes are divided into seven distinct subfamilies (ABC1, MDR/TAP, MRP, ALD, OABP, GCN20, White). This protein is a member of the ALD subfamily, which is involved in peroxisomal import of fatty acids and/or fatty acyl-CoAs in the organelle. This peroxisomal membrane protein likely plays an important role in peroxisome biogenesis. | Up-regulated by PPAR agonists |
| *Acat1* | This gene encodes a mitochondrially localized enzyme that catalyzes the reversible formation of acetoacetyl-CoA from two molecules of acetyl-CoA. Defects in this gene are associated with 3-ketothiolase deficiency, an inborn error of isoleucine catabolism characterized by urinary excretion of 2-methyl-3-hydroxybutyric acid, 2-methylacetoacetic acid, tiglylglycine, and butanone. | Up-regulated by PPAR agonists |
| *Ehhadh* | The protein encoded by this gene is a bifunctional enzyme and is one of the four enzymes of the peroxisomal beta-oxidation pathway. The N-terminal region of the encoded protein contains enoyl-CoA hydratase activity while the C-terminal region contains 3-hydroxyacyl-CoA dehydrogenase activity. Defects in this gene are a cause of peroxisomal disorders such as Zellweger syndrome. | Up-regulated by PPAR agonists |
| *Fgf21* | The protein encoded by this gene is a member of the fibroblast growth factor (FGF) family. FGF21 stimulates glucose uptake in adipocytes but not in other cell types. This effect is additive to the activity of insulin. FGF21 injection in ob/ob mice results in an increase in Glut1 in adipose tissue. FGF21 also protects animals from diet-induced obesity when overexpressed in transgenic mice and lowers blood glucose and triglyceride levels when administered to diabetic rodents. Treatment of animals with FGF21 results in increased energy expenditure, fat utilization and lipid excretion. | Up-regulated by PPAR agonists |
| *Lpcat3* | Acyltransferase which mediates the conversion of lysophosphatidylcholine into phosphatidylcholine (LPCAT activity). Catalyzes also the conversion of lysophosphatidylserine into phosphatidylserine (LPSAT activity). Has also weak lysophosphatidylethanolamine acyltransferase activity (LPEAT activity). Favors polyunsaturated fatty acyl-CoAs as acyl donors compared to saturated fatty acyl-CoAs. Is the major enzyme contributing to LPCAT activity in the liver. Lysophospholipid acyltransferases (LPLAT) catalyze the reacylation step of the phospholipid remodeling pathway also known as the Lands cycle | Up-regulated by PPAR agonists |
| *Ly6d* | May act as a specification marker at earliest stage specification of lymphocytes between B- and T-cell  development. Marks the earliest stage of B-cell specification | Up-regulated by PPAR agonists |
| *Pck2* | This gene encodes a member of the phosphoenolpyruvate carboxykinase (GTP) family. The protein is a mitochondrial enzyme that catalyzes the conversion of oxaloacetate to phosphoenolpyruvate in the presence of GTP. A cytosolic form encoded by a different gene has also been characterized and is the key enzyme of gluconeogenesis in the liver. | Up-regulated by PPAR agonists |
| *Pex1* | This gene encodes a member of the AAA ATPase family, a large group of ATPases associated with diverse cellular activities. This protein is cytoplasmic but is often anchored to a peroxisomal membrane where it forms a heteromeric complex and plays a role in the import of proteins into peroxisomes and peroxisome biogenesis. Mutations in this gene have been associated with complementation group 1 peroxisomal disorders. | Up-regulated by PPAR agonists |
| *Abcd2* | The protein encoded by this gene is a member of the superfamily of ATP-binding cassette (ABC) transporters. This protein is a member of the ALD subfamily, which is involved in peroxisomal import of fatty acids and/or fatty acyl-CoAs in the organelle. | Down-regulated by TET- like acting compounds |
| *Acsm3* | Has medium-chain fatty acid:CoA ligase activity with broad substrate specificity (in vitro). Acts on acids  from C(4) to C(11) and on the corresponding 3-hydroxy- and 2,3- or 3,4-unsaturated acids (in vitro) | Down-regulated by TET-like acting compounds |
| *Bbox1* | This gene encodes gamma butyrobetaine hydroxylase which catalyzes the formation of L-carnitine from  gamma-butyrobetaine, the last step in the L-carnitine biosynthetic pathway. Carnitine is essential for the transport of activated fatty acids across the mitochondrial membrane during mitochondrial beta-oxidation. | Down-regulated by TET- like acting compounds |
| *Bdh1* | This gene encodes a member of the short-chain dehydrogenase/reductase gene family. The encoded protein forms a homotetrameric lipid-requiring enzyme of the mitochondrial membrane and has a specific requirement for phosphatidylcholine for optimal enzymatic activity. The encoded protein catalyzes the interconversion of acetoacetate and (R)-3-hydroxybutyrate, the two major ketone bodies produced during fatty acid catabolism. | Down-regulated by TET- like acting compounds |
| *Crot* | This gene encodes a member of the carnitine/choline acetyltransferase family. The encoded protein converts 4,8-dimethylnonanoyl-CoA to its corresponding carnitine ester. This transesterification occurs in the peroxisome and is necessary for transport of medium- and long- chain acyl-CoA molecules out of the peroxisome to the cytosol and mitochondria. The protein thus plays a role in lipid metabolism and fatty acid beta-oxidation. | Down-regulated by TET- like acting compounds |
| *Cxcl12* | This gene encodes a stromal cell-derived alpha chemokine member of the intercrine family. This gene product and its receptor CXCR4 can activate lymphocytes and have been implicated in the metastasis of some cancers such as breast cancer. | Down-regulated by TET- like acting compounds |
| *Elovl2* | Condensing enzyme that catalyzes the synthesis of polyunsaturated very long chain fatty acid (C20- and  C22-PUFA). Acts specifically toward polyunsaturated acyl-CoA with the higher activity toward C20:4(n-6) acyl-CoA | Down-regulated by TET- like acting compounds |
| *Fas* | The protein encoded by this gene is a member of the TNF-receptor superfamily. This receptor contains a death domain. It has been shown to play a central role in the physiological regulation of programmed cell death, and has been implicated in the pathogenesis of various malignancies and diseases of the immune system. | Down-regulated by TET- like acting compounds |
| *Glyat* | The glycine-N-acyltransferase protein conjugates glycine with acyl-CoA substrates in the mitochondria. The protein is thought to be important in the detoxification of endogenous and xenobiotic acyl-CoA's. Two transcript variants encoding different isoforms have been found for this gene. | Down-regulated by TET- like acting compounds |
| *Hadh* | This gene is a member of the 3-hydroxyacyl-CoA dehydrogenase gene family. The encoded protein functions in the mitochondrial matrix to catalyze the oxidation of straight-chain 3-hydroxyacyl-CoAs as part of the beta-oxidation pathway. Its enzymatic activity is highest with medium-chain-length fatty acids. Mutations in this gene cause one form of familial hyperinsulinemic hypoglycemia. | Down-regulated by TET- like acting compounds |
| *Hsd17b4* | The protein encoded by this gene is a bifunctional enzyme that is involved in the peroxisomal beta-oxidation pathway for fatty acids. It also acts as a catalyst for the formation of 3-ketoacyl-CoA intermediates from both straight-chain and 2-methyl-branched-chain fatty acids. Defects in this gene that affect the peroxisomal fatty acid beta-oxidation activity are a cause of D-bifunctional protein deficiency. | Down-regulated by TET- like acting compounds |
| *Igfbp2* | Inhibits IGF-mediated growth and developmental rates. IGF-binding proteins prolong the half-life of the IGFs and have been shown to either inhibit or stimulate the growth promoting effects of the IGFs on cell culture. They alter the interaction of IGFs with their cell surface receptors | Down-regulated by TET- like acting compounds |
| *Maob* | The protein encoded by this gene belongs to the flavin monoamine oxidase family. It is an enzyme located in the mitochondrial outer membrane. It catalyzes the oxidative deamination of biogenic and xenobiotic amines and plays an important role in the metabolism of neuroactive and vasoactive amines in the central nervous sysytem and peripheral tissues. | Down-regulated by TET- like acting compounds |
| *Nox4* | This gene encodes a member of the NOX-family of enzymes that functions as the catalytic subunit the NADPH oxidase complex. The encoded protein is localized to non-phagocytic cells where it acts as an oxygen sensor and catalyzes the reduction of molecular oxygen to various reactive oxygen species (ROS). The ROS generated by this protein have been implicated in numerous biological functions including signal transduction, cell differentiation and tumor cell growth. | Down-regulated by TET- like acting compounds |
| *Onecut1* | This gene encodes a member of the Cut homeobox family of transcription factors. Expression of the encoded protein is enriched in the liver, where it stimulates transcription of liver-expressed genes, and antagonizes glucocorticoid-stimulated gene transcription. This gene may influence a variety of cellular processes including glucose metabolism, cell cycle regulation, and it may also be associated with cancer. | Down-regulated by TET- like acting compounds |
| *Slc10a2* | This gene encodes a sodium/bile acid cotransporter. This transporter is the primary mechanism for uptake of intestinal bile acids by apical cells in the distal ileum. Bile acids are the catabolic product of cholesterol metabolism, so this protein is also critical for cholesterol homeostasis. | Down-regulated by TET- like acting compounds |
| *Slc16a7* | This gene is a member of the monocarboxylate transporter family. Members in this family transport metabolites, such as lactate, pyruvate, and ketone bodies. The protein encoded by this gene catalyzes the proton-linked transport of monocarboxylates and has the highest affinity for pyruvate. | Down-regulated by TET- like acting compounds |
| *Slc25a20* | This gene product is one of several closely related mitochondrial-membrane carrier proteins that shuttle substrates between cytosol and the intramitochondrial matrix space. This protein mediates the transport of acylcarnitines into mitochondrial matrix for their oxidation by the mitochondrial fatty acid-oxidation pathway. | Down-regulated by TET- like acting compounds |
| *Slc27a2* | The protein encoded by this gene is an isozyme of long-chain fatty-acid-coenzyme A ligase family. Although differing in substrate specificity, subcellular localization, and tissue distribution, all isozymes of this family convert free long-chain fatty acids into fatty acyl-CoA esters, and thereby play a key role in lipid biosynthesis and fatty acid degradation. It is expressed primarily in liver and kidney, and is present in both endoplasmic reticulum and peroxisomes, but not in mitochondria. | Down-regulated by TET- like acting compounds |
